# Blockade of the AHR restricts a Treg-macrophage suppressive axis induced by L-Kynurenine

**DOI:** 10.1038/s41467-020-17750-z

**Published:** 2020-08-11

**Authors:** Luis Felipe Campesato, Sadna Budhu, Jeremy Tchaicha, Chien-Huan Weng, Mathieu Gigoux, Ivan Jose Cohen, David Redmond, Levi Mangarin, Stephane Pourpe, Cailian Liu, Roberta Zappasodi, Dmitriy Zamarin, Jill Cavanaugh, Alfredo C. Castro, Mark G. Manfredi, Karen McGovern, Taha Merghoub, Jedd D. Wolchok

**Affiliations:** 1 Swim Across America and Ludwig Collaborative Laboratory, Immunology Program, Parker Institute for Cancer Immunotherapy, Memorial Sloan Kettering Cancer Center, New York, NY USA; 2 Immuno-Oncology Service, Human Oncology and Pathogenesis Program, Memorial Sloan Kettering Cancer Center, New York, NY USA; 3 Ikena Oncology, Boston, MA USA; 4 Department of Neurology and Louis V. Gerstner Jr Graduate School of Biomedical Sciences, Memorial Sloan Kettering Cancer Center, New York, NY 10065 USA

**Keywords:** Cancer microenvironment, Immunosuppression, Tumour immunology

## Abstract

Tryptophan catabolism by the enzymes indoleamine 2,3-dioxygenase 1 and tryptophan 2,3-dioxygenase 2 (IDO/TDO) promotes immunosuppression across different cancer types. The tryptophan metabolite L-Kynurenine (Kyn) interacts with the ligand-activated transcription factor aryl hydrocarbon receptor (AHR) to drive the generation of Tregs and tolerogenic myeloid cells and PD-1 up-regulation in CD8^+^ T cells. Here, we show that the AHR pathway is selectively active in IDO/TDO-overexpressing tumors and is associated with resistance to immune checkpoint inhibitors. We demonstrate that IDO-Kyn-AHR-mediated immunosuppression depends on an interplay between Tregs and tumor-associated macrophages, which can be reversed by AHR inhibition. Selective AHR blockade delays progression in IDO/TDO-overexpressing tumors, and its efficacy is improved in combination with PD-1 blockade. Our findings suggest that blocking the AHR pathway in IDO/TDO expressing tumors would overcome the limitation of single IDO or TDO targeting agents and constitutes a personalized approach to immunotherapy, particularly in combination with immune checkpoint inhibitors.

## Abstract (web summary)

The tryptophan metabolite kynurenine is an endogenous ligand of the aryl hydrocarbon receptor (AHR). Here, the authors show that AHR targeting in IDO/TDO-expressing tumours counteracts a regulatory T cell/macrophage suppressive axis and synergizes with immune checkpoint blockade to hinder tumour growth.

## Introduction

The catabolism of the essential amino acid Tryptophan (Trp) into Kynurenine (Kyn) metabolites is a central pathway maintaining peripheral tolerance to immune-privileged sites (eye, testis, and placenta), together with the adenosine/purinergic pathway and with immune checkpoints, such as CTLA-4 and PD-1^1^. Expression of indoleamine 2,3-dioxygenase 1 (IDO1), or tryptophan 2,3-dioxygenase 2 (TDO2)—hereafter referred to IDO/TDO—by tumors has been shown to act as a driver of immune suppression through myeloid-derived suppressor cell (MDSCs) recruitment and activation^2^, T-cell anergy^3^ and is associated with poor clinical prognosis in glioblastoma^4^ or acquired resistance mechanism to PD-1 and CTLA-4 blockade in pre-clinical models^2,5,6^. Several efforts have been directed to blocking the IDO pathway. More recently the inhibition of IDO pathway among unselected patient populations in a large Phase III trial (ECHO-301/KEYNOTE-252) did not improve the therapeutic outcome to PD-1 blockade^7^, but highlight the need for further studies aimed at investigating the mechanisms of metabolic dysregulation via Trp catabolism in antitumor immunity.

Recent research has revealed that the IDO/TDO product Kyn can act as a key signaling molecule through activation of the ligand-activated transcription factor aryl hydrocarbon receptor (AHR), which is implicated in a variety of biochemical processes including control of the immune response^8–10^. The production of Kyn and its metabolites by TDO was shown to reach concentrations sufficient to activate the AHR pathway in the tumor microenvironment (TME) of a glioblastoma model^4^. Recent studies demonstrated that Kyn can be found in high concentrations in the plasma of advanced-stage cancer patients and a high serum Kyn/Trp ratio correlates with poor prognosis after PD-1 blockade in several cancer types, including lung cancer, melanoma, and renal cell carcinomas^11–13^, while systemic Kyn depletion with a PEGylated Kyn-degrading enzyme led to cancer control in pre-clinical settings^14^. In the present study, we identify AHR pathway activity, a downstream component of IDO/TDO-Kyn axis, as a common feature of cancers expressing IDO or TDO and as a driver of T cell dysfunction by promoting a Treg-macrophage suppressive axis in the TME. We show that targeting the AHR in tumors with an active Trp catabolic pathway overcomes immunosuppressive mechanisms and sensitize tumors to anti-PD-1 therapy. Our findings provide a rationale for assessing active metabolic pathways in tumors, in a precision-medicine type of approach, and exploring AHR inhibition in combination with other immunotherapy strategies.

## Results

### AHR expression is associated with immunosuppression in human tumors

**Fig. 1:**
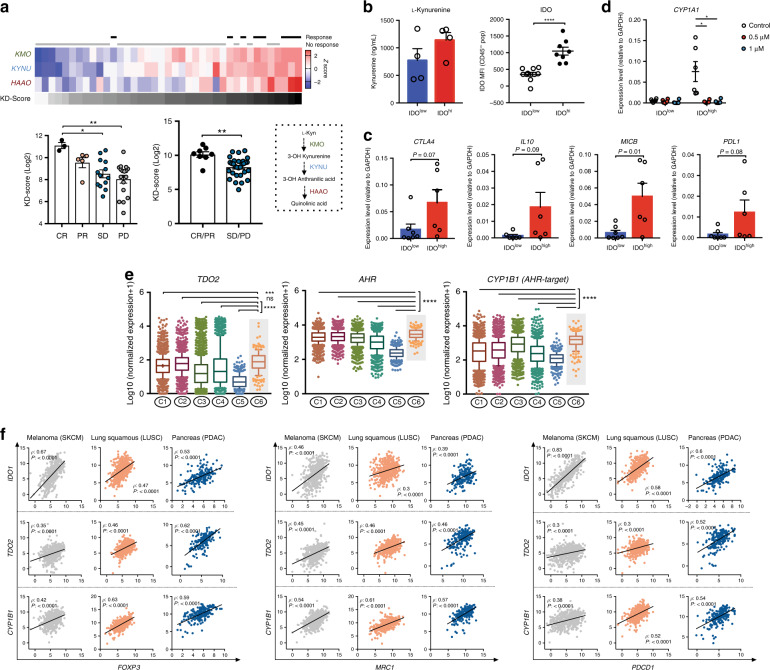
An active IDO/TDO-Kyn-AHR pathway associates with immune suppressive features in human cancers. **a** Top: heat-map representing the gene-expression analysis of Kyn-degrading enzymes (*KMO, KYNY, HAAO*) and KD-Score (grayscale) in responsive (complete response/CR or partial response/PR) highlighted in black (*n* = 8), or non responsive (stable disease/SD or progressive disease/PD) highlighted in gray (*n* = 31), melanoma patients after PD-1 blockade. Bottom: bar graph with quantification of KD-Score. **b** ELISA quantification of L-Kynurenine in blood serum of melanoma patients (left) categorized as IDO^high^ or IDO^low^ (*n* = 4 IDO^high^ and *n* = 4 IDO^low^) based on intracellular IDO staining by FACS of melanoma cell suspensions (right) (*n* = 8 IDO^high^ and *n* = 10 IDO^low^). **c** mRNA of immunoregulatory markers by qRT-PCR analysis in IDO^high^ and IDO^low^ melanoma cell suspensions. **d** mRNA of AHR-target genes *CYP1A1* and *CYP1B1* by qRT-PCR analysis in IDO^high^ and IDO^low^ melanoma cell suspensions after treatment with selective AHR inhibitor KYN-101 for 24 h (*n* = 6 IDO^high^ and *n* = 6–7 IDO^low^). **e** Distribution of log-transformed expression levels of AHR-related genes (*TDO2, AHR,* and *CYP1B1*) across six immune subtypes of cancer (C1: wound-healing, C2: IFN-γ dominant, C3: inflammatory, C4: lymphocyte-depleted, C5: immunologically quiet, C6: TGF-β dominant). Data plotted as box and whiskers with the median and limits within the 10–90% percentile. **f** Correlation analysis between Treg marker (*FOXP3*), myeloid-cell marker (*MRC1/CD206*), inhibitory checkpoint (*PDCD1/PD-1*), and AHR-related genes *IDO1, TDO2*, and *CYP1B1* in TCGA RNAseq data of skin melanoma (SKCM), squamous lung (LUSC) and pancreatic adenocarcinoma (PDAC) analyzed by Spearman rank correlation. Data shown are represented as mean values ± SEM with two-tailed unpaired Student’s *t* test in (**a**–**d**), one-way ANOVA test with Tukey correction in (**a**) and Kruskal–Wallis with Dunn correction in (**e**). *P* value: *P < 0.05, **P < 0.01, ***P < 0.001, ****P < 0.0001.

To address whether the IDO/TDO-Kyn-AHR axis limits the efficacy of immune-based therapies, we examined an RNAseq dataset of tumors from 68 patients with advanced melanoma before and after anti-PD-1 therapy^15^. We found an enrichment in the expression of enzymes involved in Kyn degradation, such as kynurenine 3-monooxygenase (*KMO*), kynureninase (*KYNU*) and 3-Hydroxyanthranilic acid dioxygenase (*HAAO*), in patients who derived clinical benefit from PD-1 blockade (Fig. 1a) in comparison to those who did not (*P* = 0.001). We further investigated the potential role of AHR in promoting well-defined immune suppressive mechanisms. Using single-cell suspensions prepared from malignant melanoma resections banked at MSK and classified as low or high based on their intracellular expression of IDO (Fig. 1b), we detected higher levels of immune-regulatory factors (*IL-10, PD-L1,* and *CTLA-4*) in IDO^high^ samples compared to IDO^low^ samples (Fig. 1c). Furthermore, we detected higher levels of the AHR-target genes cytochrome P4501A1 (*CYP1A1*) and cytochrome P4501B1 (*CYP1B1*) in IDO^high^ samples versus IDO^low^ samples. Increases in the AHR-target genes were reversed upon treatment with a selective AHR inhibitor in vitro (KYN-101) (Fig. 1d).

Using the expression of *CYP1B1* as a surrogate of AHR activity, we found its association with poorer overall survival in renal and urothelial cancer patients (Supplementary Fig. 1a). In addition, *CYP1B1* expression was positively correlated with both *IDO1* and *TDO2* expression in skin melanoma (SKCM), squamous lung carcinoma (LUSC), diffuse large B-cell lymphoma (DLBC) and pancreatic adenocarcinoma (PDAC) (Supplementary Fig. 1b). These obeservations suggest the existence of IDO/TDO-mediated activation of the AHR pathway across cancer types. Using a recently described classification of tumors based on their immunogenomic profile^16^, we found an upregulated expression of Kyn-AHR pathway-related genes (*TDO2, AHR,* and *CYP1B1*) within tumors presenting a TGF-β–dominant immune signature (C6 in Fig. 1e). Such signature has been reported as the one predicting the poorest prognosis and associated with a myeloid dominated microenvironment, with low lymphocytic infiltrate and high on M2-TAMs content on their constituent tumors^16^. Expression of *TDO2*, *AHR*, and *CYP1B1* was the highest in the TGF-β–dominant immune subtype (C6), while *IDO1* was found highest in the interferon gamma (IFN-γ) dominant signature (C2) followed by C6, likely in reflection to its IFN-inducible nature^17^. Furthermore, TCGA RNAseq analysis revealed a strong correlation between AHR-pathway related genes (*IDO1, TDO2,* and *CYP1B1*) and myeloid-cell markers (*MRC1/CD206, CD14*), Treg markers (*FOXP3, IL2RA*) and inhibitory checkpoints (*PDCD1, CTLA-4*) (Fig. 1e and Supplementary Fig. 1c). Taken together, these findings suggest that activation of the Kyn-AHR pathway is a common feature of IDO or TDO-expressing cancers and may provide a novel immunoregulatory mechanism associated with resistance to checkpoint inhibition.

### Immune dysfunction is a feature of IDO/TDO-expressing tumors

**Fig. 2:**
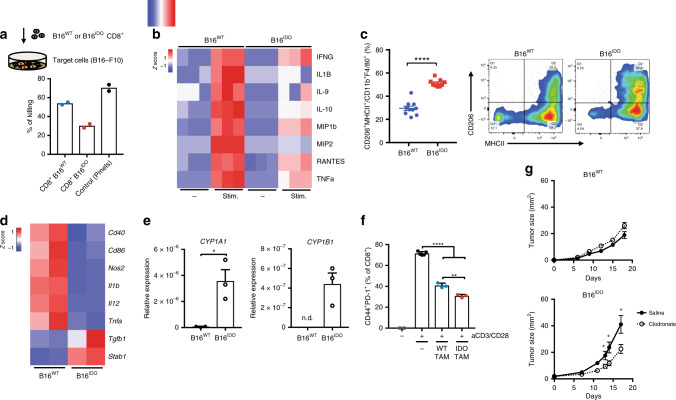
IDO-overexpressing melanomas present a dysfunctional myeloid-enriched immune landscape. **a** Top: schematic representation of the experimental setup for the 3D collagen-fibrin gel killing assay and bottom, quantification of target cell killing by tumor-isolated CD8^+^ T cells or tumor-antigen (gp100)-specific CD8^+^ T cells (pmels) (Data is representative of two independent experiments). **b** Multiplex analysis of cytokines from ex vivo tumor-isolated CD8^+^ T cells stimulated with aCD3/CD28 beads for 48 h. **c**, flow cytometric analysis and quantification of M2-like TAMs (CD206^+^MHCII^+^ in CD11b^+^F4/80^+^) cell populations in B16^IDO^ or B16^WT^ tumors at day 14 post implantation (*n* = 10 (B16^WT^) and *n* = 9 (B16^IDO^)). Results are representative of three independent experiments. **d** Heat-map of mRNA expression of M1 and M2 markers and **e**, of AHR-target genes in TAMs as determined by RT-PCR, data were relative to *GAPDH* expression and z-score normalized (*n* = 3). **f** in vitro suppressive activity of TAMs purified from B16^IDO^ or B16^WT^ tumor-bearing mice at day 14 post implantation in CD8^+^ T cell activation state at a ratio of 1:2 CD8^+^ to TAMs (*n* = 3). **g** Tumor progression of B16^IDO^ or B16^WT^ implanted in mice treated with clodronate liposome (1 mg/mouse) (*n* = 8) or control (*n* = 8). Results are representative of two independent experiments. Data is represented as mean values ± SEM. Two-tailed unpaired Student’s *t* test in (**c**–**e**, **g**) and one-way ANOVA test with Tukey correction in (**f**). *P* value: *P < 0.05, **P < 0.01, ***P < 0.001, ****P < 0.0001.

To further investigate the role of the Kyn-AHR pathway in promoting cancer-associated immune suppression in a more controlled setting, we generated a pre-clinical model of B16-F10 melanoma overexpressing IDO (B16^IDO^) or TDO (B16^TDO^). These models allowed us to isolate the effects of IDO or TDO expression by comparing it to the parental cell line (B16^WT^), which expresses minimal levels of the two enzymes. Increased production of Kyn was detected in B16^IDO^ and B16^TDO^ compared to B16^WT^ (Supplementary Fig. 2a). We found no differences in the tumor growth rate of B16^IDO^ and B16^TDO^ tumors (Supplementary Fig. 2b) when implanted in immunodeficient Rag^−/−^ or WT immunocompetent mice. In contrast, a faster tumor growth is observed in B16^WT^ tumors in Rag^−/−^ comparison to WT. These finding suggest that T cells play a role at controlling tumor progression in the B16^WT^ models, but not B16^IDO/TDO^. We hypothesized that an immunosuppressive TME induced by IDO/TDO overexpression could promote a dysfunctional T cell effector function. Therefore, we next investigated the extent of altered tumor immunity in the context of Kyn-AHR pathway activation to further delineate the mechanisms underlying IDO-induced immune suppression using the B16^IDO^ model. Gene expression analysis of whole-tumors revealed a distinct immune signature in B16^IDO^ tumors, with reduced expression level of Th1 genes, including *IFNG, IL1B, TNFA, CD40, CD86* and *GZMB* (Supplementary Fig. 2c). Multiplex analysis of intratumoral cytokines revealed reduced levels of T cell-related cytokines, such as IFNγ, CXCL9, CCL5/RANTES and IL-15, but increased VEGF, a known AHR-responsive gene^18^ (Supplementary Fig. 2d). To assess the tumoricidal functional state of intratumoral CD8^+^ T cells, we sorted CD8^+^ T cells from IDO-overexpressing tumors by fluorescence activated cell sorting (FACS) and further assessed them in a 3D collagen-fibrin-based killing assay, as previously described^19^. Analysis of the killing ability of effector CD8^+^ T cells revealed a dysfunctional phenotype, with a 30% killing efficiency of B16^IDO^-isolated CD8^+^ T cells as compared to 54% in B16^WT^-isolated CD8^+^ T cells (*P* = 0.01) and 70% of control in vitro activated tumor antigen-specific CD8^+^ T cells (pmel1.1), which recognize the melanosomal tumor antigen gp100 (pmel1.1) (*P* = 0.009) (Fig. 2a). In addition, supernatants from CD8^+^ T cells isolated from B16^IDO^ tumors contained lower levels of effector T cell cytokines, including IFNγ, IL-1β, RANTES, and TNFɑ after restimulation with anti-CD3/CD28 as compared to B16^WT^-isolated CD8^+^ T cells (Fig. 2b). Similar dysfunctional phenotype was observed in CD8^+^ T cells of the B16^TDO^ model (Supplementary Fig. 2e) Taken together, our results support a causal role for the expression of Trp-catabolic enzymes in driving an adaptive immune resistance mechanism, in part through inhibition of CD8^+^ T cells cytolytic function.

Further analysis of the immune-infiltrate in B16^IDO^ tumors revealed an enrichment of myeloid cells, with increased abundance of TAMs (CD11b^+^F4/80^high^Ly6G^−^) and CD11c^+^ dendritic cells (DCs) (CD11c^+^MHCII^+^) in comparison with B16^WT^ tumors (Fig. 2c, Supplementary Fig. 3a). Similar phenotype was observed in the B16^TDO^ model (Supplementary Fig. 2e) and other cancer cell line models expressing high levels of IDO in baseline conditions, such as the breast carcinoma 4T1^2^ and colorectal cancer model MC38^20^. Similar to a recent report linking the AHR with a suppressive state of TAMs in a glioblastoma model^21^, tumor cell overexpression of IDO promoted an immunoregulatory M2-like phenotype in TAMs. B16^IDO^ TAMs were more frequent and presented increased expression of the mannose receptor CD206/MRC1, an M2-marker (Fig. 2c, Supplementary Fig. 3b). Gene-expression analysis of B16^IDO^-isolated TAMs showed reduced expression levels of M1-markers (*NOS2, IL12, TNFa, CD86, CD40*) and upregulation of the M2-markers *STAB1* and *TGFB1* (Fig. 2d) compared to B16^WT^. TAMs accumulated over time as tumors progressed while retaining their M2-status (Supplementary Fig. 3c) and IDO overexpression promoted a higher frequency of TGF-β-expressing DCs (*P* = 0.02) relative to control (Supplementary Fig. 3d), consistent with a tolerogenic phenotype^22^. Immunophenotyping analysis of B16^IDO^ and B16^TDO^ tumors revealed an upregulation of AHR expression in tumor-infiltrating myeloid-cell populations, such as Ly6C^+^ Monocytes (CD11b^+^Ly6C^high^Ly6G^−^), conventional DCs (CD11b^+^CD11c^+^), polymorphonuclear cells (CD11b^+^Ly6G^+^Ly6C^low^) and particularly TAMs in comparison to B16^WT^ tumors (Supplementary Fig. 3e). Gene-expression analysis revealed an upregulated expression of the *CYP1A1* and *CYP1B1* genes in B16^IDO^ TAMs, indicating engagement of the AHR pathway (Fig. 2e). In addition, we found their more potent T cell inhibitory function than TAMs isolated from B16^WT^, as evidenced by their ability to modulate the expression of activation markers (CD44, PD1) in autologous CD8^+^ T cells in vitro (Fig. 2f).

Previous studies revealed that the presence of immunosuppressive cell types, including MDSCs and M2-like TAMs, plays a critical role in limiting responses to ICB^23,24^, while their depletion enhances antitumor immunity^25,26^. We therefore investigated the potential interplay between Kyn-AHR pathway and myeloid cell-mediated T cell inhibition. Tumor progression rate of B16^IDO^ tumors was delayed in AHR deficient mice (AHR^KO^) (Supplementary Fig. 3f) and depletion of macrophages with αCSF1-R (Supplementary Fig. 3g–h) or clodronate liposomes (Fig. 2g) delayed the progression of IDO-expressing tumors but not wild-type tumors. Tumor progression delay after macrophage depletion was reversed upon CD8^+^ T cell depletion (Supplementary Fig. 3i), suggesting that TAMs support tumor progression primarily by inhibiting CD8^+^ T cell immunity.

To further assess the effects of Kyn-mediated activation of AHR on macrophage effector function, we cultured Kyn-treated bone marrow-derived antigen-presenting cells and observed decreased expression of the antigen-presenting machinery proteins MHC Class I and II, and the co-stimulatory molecule CD86 (Supplementary Fig. 4a). This effect was reversed upon treatment with the AHR inhibitor CH-223191 (Supplementary Fig. 4b). In addition, higher levels of certain inflammatory cytokines, including IL-1β, IL-6, and VEGF, were observed in the culture media after treatment with Kyn in a dose-dependent manner (Supplementary Fig. 4c). To assess AHR-mediated changes in antigen presentation function by APCs, which could contribute to the impaired immunity in the IDO/TDO-expressing models, we co-cultured carboxyfluorescein succinimidyl ester (CFSE)-labeled pmel1.1 CD8^+^ T cells (pmels) with gp100-loaded BM-APCs pre-exposed to increasing concentrations of Kyn. We observed a significant decrease in the proliferation rate (CFSE^low^) and activation state (CD44, GzmB) of pmels cultured in the presence of AHR-activated BM-APCs in a dose-dependent manner (Supplementary Fig. 4d). Culture of pmels with AHR^KO^ BM-APCs increased their activation profile (% of CD44^+^CD25^+^) in comparison to control (Supplementary Fig. 4e) while AHR^KO^ BM-APCs presented a less immunoregulatory phenotype, with downregulation of PD-L1 and CD206 (Supplementary Fig. 4f). Taken together, our findings demonstrate that Kyn-mediated activation of AHR is capable of impairing the effector function of macrophages and antitumor T cell immunity.

### Kyn-AHR signaling promotes Treg and TAM cooperative effects

**Fig. 3:**
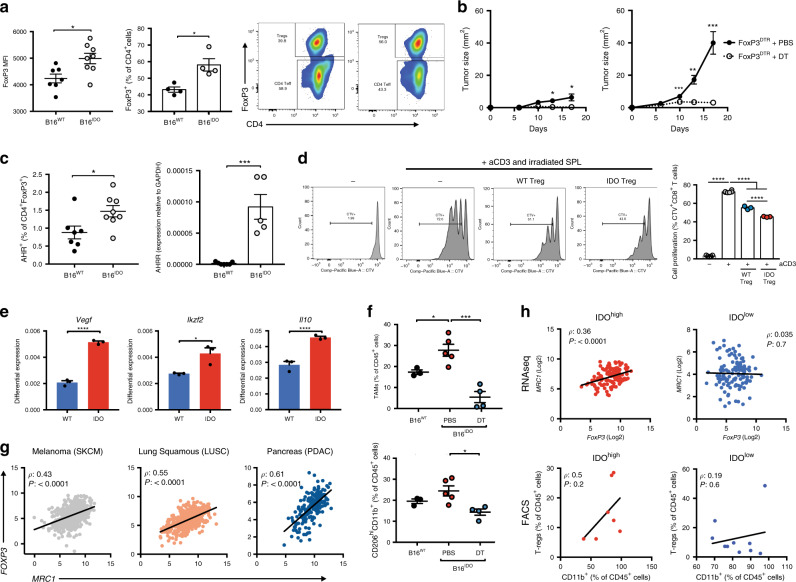
FoxP3^+^T cells co-operate with TAMs in promoting immune suppression of IDO + tumors. **a** Flow cytometric analysis of FoxP3 expression in tumor-infiltrating CD4^+^ populations (left), and of Tregs (CD4^+^FoxP3^+^) (right) (*n* = 7 (B16^WT^) and *n* = 8 (B16^IDO^)). Results representative of three independent experiments. **b** Tumor progression of B16^WT^ (left) and B16^IDO^ (right) tumors implanted in FoxP3^DTR^ mice treated with PBS or DT (1 μg/mouse) (*n* = 5 per group). **c** FACS analysis of AHR expression (left) (*n* = 7 (B16^WT^) and *n* = 8 (B16^IDO^)) and mRNA analysis by qRT-PCR analysis of *AHRR* expression in B16^IDO^ and B16^WT^ tumor-isolated T-regs (right). **d** In vitro suppressive activity of tumor-isolated Tregs isolated from B16^IDO^ or B16^WT^-bearing FoxP3^GFP^ mice. Left: Representative histograms of CD8^+^ T cell proliferation at a ratio of 1:1 (Effectors:Targets). Right: FACS quantification of T cell proliferation (CTV^low^) (*n* = 3). **e** mRNA of Treg markers by qRT-PCR analysis in tumor-isolated Tregs from B16^WT^ and B16^IDO^ tumors (*n* = 3). **f** FACS analysis of TAMs (top) and M2-like myeloid populations (CD206^high^CD11b^+^) (bottom) in B16^IDO^ tumors after Treg depletion in FoxP3^DTR^ mice injected with DT (1 μg/mouse) or PBS (B16^WT^*n* = 3; B16^IDO^ PBS *n* = 4, DT *n* = 5 mice/group). **g** Correlation analysis between myeloid marker (MRC1/CD206) and Treg marker (FoxP3) in TCGA RNAseq data of of skin melanoma (SKCM), squamous lung (LUSC) and pancreatic adenocarcinoma (PDAC). **h** Correlation analysis in TCGA RNAseq data of IDO^high^ and IDO^low^ skin melanomas (SKCM) analyzed by Spearman rank correlation. Bottom: correlation analysis between frequency of Tregs (CD4^+^FoxP3^+^) and myeloid cells (CD11b^+^) in FACS analyzed IDO^high^ and IDO^low^ melanoma patients’ biopsies by Spearman rank correlation. Results in (b–f) are representative of two independent experiments. Data is represented as mean values ± SEM. Two-tailed unpaired Student’s *t* test was used when only two groups were compared, and one-way ANOVA was applied with comparison of more than two groups. *P* value: *P < 0.05, **P < 0.01, ***P < 0.001, ****P < 0.0001.

Consistent with recent reports^2^ and our previous results (Fig. 1 and Supplementary Fig. 1C), IDO-expressing tumors presented an increased Treg frequency and upregulated expression of FoxP3 in intratumoral CD4^+^ T cells from in relation to B16^WT^ (Fig. 3a). The Kyn-AHR pathway was previously implicated in epigenetic regulation of FoxP3^27^ and in the generation of Tregs^28^. In our pre-clinical model, Tregs seem to play a major role in mediating B16^IDO^ tumor progression given that the selective depletion of Tregs, using a transgenic mouse strain engineered with a transgene that expresses the diphtheria toxin receptor (DTR) under control of the Foxp3 promoter (Foxp3^DTR^ mice), led to regression of B16^IDO^ tumor growth (Fig. 3b). B16^IDO^ Tregs had increased expression of AHR and the AHR-target AHR repressor gene (*AHRR*) (*P* = 0.0003), as compared to WT Tregs (Fig. 3c). Tregs isolated from B16^IDO^ tumors, implanted in transgenic mice expressing Foxp3-GFP fusion protein (Foxp3^GFP^ knock-in mice), were superior in their ability to suppress the proliferation and activation of of autologous CD8^+^ T cells in vitro when compared with B16^WT^-isolated Tregs (Fig. 3d). On the transcriptional level, Tregs isolated from the B16^IDO^ tumors exhibited a more prominent expression of the inhibitory factors such as *IL-10, VEGF, CTLA-4*, as well as upregulation of *Ikzf2* (Helios), *OX40*, and *Ki67* (Fig. 3e). Interestingly, bead array-based analysis of culture supernatants from B16^IDO^ Tregs stimulated with anti-CD3/CD28 revealed increased levels of the chemokines MIP-1 alpha, MIP-1 beta, RANTES and of IFNγ (Supplementary Fig. 5a).

Specific depletion of Tregs using Foxp3^DTR^ mice significantly abrogated the myeloid-enriched phenotype found in B16^IDO^ tumors, with reduced infiltration of CD11b^+^ cells and M2-like TAMs after treatment with DT (Fig. 3f). These data suggest that Tregs may promote a protumoral TAM activity, similar to what described in other recent reports^29,30^. Consistently, RNAseq analysis of human cancers revealed a strong positive correlation between the expression of Treg markers (*FOXP3, IL2RA*) and myeloid cell markers (*MRC1, CD14,* and *ITGAM*) across several tumor types (Fig. 3g). In melanoma, the correlation was particularly strong in tumors expressing high levels of IDO (IDO^high^) and non-existent in those expressing low levels of IDO (IDO^low^), with similar observations when categorizing tumors by *CYP1B1* expression (Fig. 3h, Supplementary Fig 5b). Accordingly, a similar correlation between Tregs (CD4^+^FoxP3^+^) and CD11b^+^ cells was detected in IDO^high^ melanoma cell suspensions as compared to IDO^low^ samples (Fig. 3h). With that, we hypothesized that an interplay between Tregs and TAMs exist, with the Kyn-AHR signaling playing a role in promoting such immunoregulatory axis. To assess if Tregs could induce changes in the activation state of myeloid cells through the activation of AHR, we made use of Treg-myeloid cell co-culture models. Pre-treatment of ex vivo splenic Tregs isolated from Foxp3^GFP^ hosts with Kyn led to expansion of M2-like macrophages (CD206^+^ and PD-L1^high^) in co-cultures. The expansion of the M2-like macrophages was reversed with selective AHR inhibitor (CH-223191) (Supplementary Fig 5c). Moreover, AHR^KO^ Tregs presented diminished inhibitory function over T cell activation and the engagement of AHR with increasing concentrations of Kyn in WT Tregs, but not AHR^KO^ Tregs, led to superior suppressive function over T cell priming by BM-APCs (Supplementary Fig 5d–e). Taken together, our data suggest that an engagement of the AHR pathway by IDO or TDO-derived Kyn can drive a Treg-macrophage suppressive axis and tumor progression.

### AHR blockade reverses IDO/TDO-mediated immunosuppression

**Fig. 4:**
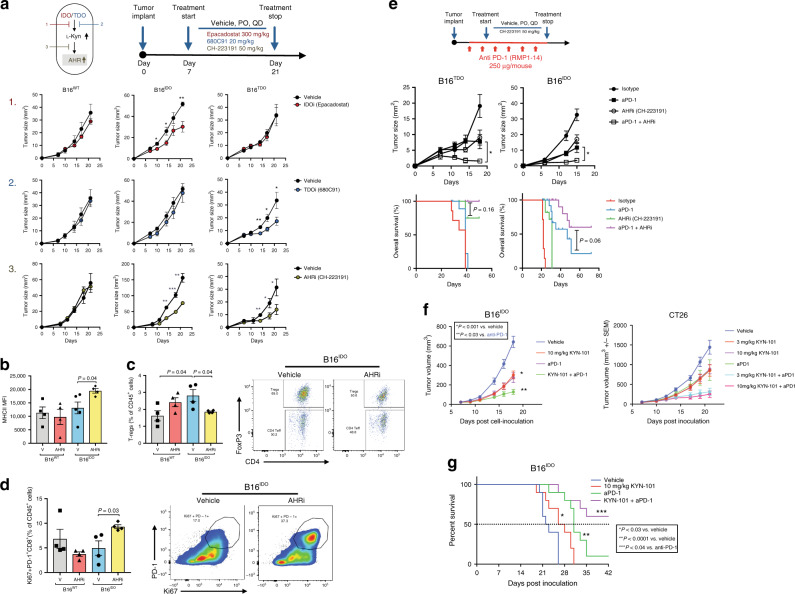
Selective AHR inhibition reverses IDO/TDO-mediated tumor progression and improves the efficacy of PD-1 blockade. **a** Top left: scheme figure with experimental approach of targeting multiple steps of Trp-catabolic pathway. Top right: therapy regimen (PO, QD). Bottom: tumor progression of orthotopically injected B16^WT^, B16^IDO^ and B16^TDO^ tumors in mice treated with vehicle or inhibitors (Epacadostat/IDO inhibitor 300 mg/kg/d, 680C91/TDO inhibitor 20 mg/kg/d, CH-223191/AHR inhibitor 50 mg/kg/d). (*n* = 5 mice per group for two independent experiments). **b** FACS analysis of MHCII expression (*n* = 4 per group, *n* = 5 B16^IDO^ vehicle). **c** Quantification of frequency and representative plots of intratumor Tregs (CD4^+^FoxP3^+^CD25^+^/% of CD45^+^) and **d**, quantification of frequency and representative plots of Ki67^+^PD-1^+^ CD8^+^ T cells from B16^WT^ and B16^IDO^-bearing mice treated with vehicle or AHR inhibitor (CH-223191) (*n* = 4 per group). **e** Top: therapy regimen. bottom: mean tumor size and overall survival of B16^IDO^ and B16^TDO^ tumor-bearing mice treated with AHR inhibitor (CH-223191), anti-PD-1 alone or in combination with AHR inhibitor (combo) (*n* = 5 B16^TDO^ and *n* = 10 B16^IDO^). **f** Mean tumor size of B16^IDO^ and CT26 and **g**, overall survival of B16^IDO^ tumor-bearing mice treated with the optimized AHR inhibitor (KYN-101), anti-PD-1 alone or in combination with AHR inhibitor (combo) (*n* = 10 mice per group). Results are representative of two independent experiments. Data represented as mean values ± SEM. Two-tailed Student’s *t* test was used when only two groups were compared and log-rank (Mantel–Cox) test was used for survival comparison. *P* value: *P < 0.05, **P < 0.01, ***P < 0.001, ****P < 0.0001.

Based on these findings, we sought to explore the effects of using selective AHR inhibitors in controlling tumor growth. Since cancer cells can preferentially up-regulate IDO or TDO, and the release of Kyn is downstream of both pathways, we reasoned that pharmacologic AHR inhibition might lead to superior immune effects, compared with selective IDO or TDO inhibition strategies in controlling IDO/TDO-expressing tumor growth by ensuring that both downstream pathways are blocked (Fig. 4a). Treatment with Epacadostat, a selective inhibitor of IDO, led to inhibition of growth of B16^IDO^, but not of B16^WT^ or B16^TDO^ tumors, while 680C91, a specific TDO inhibitor, led to delayed progression of B16^TDO^, but not of B16^WT^ or B16^IDO^ tumors. In contrast, treatment with an AHR inhibitor (AHRi1, CH-223191) led to tumor growth inhibition in both B16^IDO^ or B16^TDO^ models, but not B16^WT^ (Fig. 4a). Similar results were observed using clofazimine, a recently described antagonist of the AHR^31^, and KYN-101, an optimized, selective synthetic antagonist of AHR (Supplementary Fig. 6a). KYN-101 shares pharmacological properties with the AHR antagonist selected for clinical development in cancer patients. The clinical candidate is similar in potency, selectivity and in vivo activity to that demonstrated with KYN-101 (Supplementary Fig. 7). Gene-expression analysis of the whole tumors after AHRi1 treatment revealed upregulation of *GZMB, CD86*, and *IFNG* in B16^IDO^ tumors in comparison to control (Supplementary Fig 6b) which is consistent with a previous report implicating AHR signaling in shaping IFN-mediated responses^32^. We also found that the treatment of B16^IDO^ tumors with AHRi1 in immunodeficient mice (Rag^−/−^) did not completely abolish its therapeutic efficacy, suggesting that a non-T cell TME component is potentially involved in the antitumor effect of AHR inhibitors (Supplementary Fig. 6c).

Immunophenotypic analysis of B16^IDO^ tumors after AHRi1 treatment revealed repolarization of TAMs, as characterized by increased MHCII expression (Fig. 4b), and an increase in antigen-specific T cell priming. AHRi1 treatment resulted in an increase in adoptively transferred pmels and their activation profile within tumors and tumor-draining lymph nodes (TDLN) of B16^IDO^-bearing mice (Supplementary Fig. 6d). In addition, treatment with AHRi1 led to decreases in the frequency of intratumoral Tregs (Fig. 4c). Gene-expression analysis of intratumoral Tregs and effector CD4+ T cells (Teff) revealed a markedly differential gene-expression signature after AHRi1 treatment, consistent with a dysfunctional and less suppressive state in Tregs and Th-1-like effector phenotype in Teff in both B16^IDO^ and B16^TDO^ models (Supplementary Fig. 8a–b).

We found that intratumoral CD8^+^ T cells from the AHRi1 group had increased expression of the proliferation marker Ki67, the transcription factor T-bet (Supplementary Fig. 8c) and were enriched in PD-1^+^Ki67^+^ cells (Fig. 4d), which has been recently linked with a T cell reinvigoration phenotype^33^. In addition, we observed an increased population of CD8^+^ T cells with a marked increased in a double-positive Eomes^+^T-bet^+^ cells (Supplementary Fig. 8d), that was previously described as characteristic of a reversibly exhausted population^33^. Similar immunomodulatory effects of AHRi1 treatment were observed in the B16^TDO^ model (Supplementary Fig. 9). FACS sorting of intratumoral CD8^+^ T cells from B16^IDO^-bearing mice after treatment with AHRi1 revealed increased expression of pro-inflammatory cytokines (IFN-γ, IL-10, MIP1a, and MIP1b) after ex vivo stimulation (Supplementary Fig. 8e). Given the immune modulatory effects of AHR inhibition in the TME of IDO/TDO-expressing models, we next evaluated if its combination with immune checkpoint blockade (anti-PD-1) could yield additional antitumor activity. Use of AHRi1 (CH-223191) with anti-PD-1 led to delayed tumor progression and prolonged survival in B16^IDO^ and B16^TDO^ tumors models when compared with single agent therapy (Fig. 4e). On the other hand, addition of AHRi1 did not improve the response to anti-PD-1 alone in a model lacking IDO/TDO-Kyn pathway activation (B16^WT^) (Supplementary Fig. 10a). In addition, tumor-free survivors in the combination therapy group were resistant to tumor re-implantation, indicating the development of long lasting adaptive immunity (Supplementary Fig. 10b). Combination of AHRi and anti-PD-1 increased markers of activation in intratumoral CD8^+^ T cells (CD44, GrzB and Ki67) and myeloid cells (MHCII, PD-L1) (Supplementary Fig. 10c–d). Interestingly, an optimal efficacy of AHRi and PD-1 blockade was dependent on the presence of myeloid cells (monocytes/macrophages), as their depletion using αCSF1R or using CCR2^KO^ mice attenuated the efficacy of the combination AHRi1+ anti-PD-1 therapy (Supplementary Fig. 10e).

Furthermore, we found that the combination of KYN-101 (AHRi2) and anti-PD-1 led to improved tumor growth delay and extended survival in B16^IDO^ (Fig. 4f, g). Similar oberservation was made in the CT26 colorectal cancer model expressing endogenous high levels of IDO (Fig. 4f). Thus, these data indicate that the reversion of a regulatory phenotype in immune cells can be achieved by selective pharmacologic targeting of the AHR to promote more effective antitumor immunity and enhance the effects of PD-1 blockade.

## Discussion

Upregulation of IDO, together with other inhibitory checkpoint molecules such as PD-L1, LAG-3, and VISTA, are well-documented mechanisms of acquired resistance to immunotherapy^24^. A high frequency of IDO and TDO expression (32 and 35% respectively^34^) is described in multiple cancer types, such as melanoma, lung cancer, pancreatic cancer and renal cell carcinoma^35^. Given the mouting evidence that high IDO expression can lead to inhibition of the effector function of T cells, either directly or indirectly through non-T cell components^36,37^, strategies to effectively target this metabolic pathway can reverse tumor immune resistance mechanisms. Interestingly, increases in the IDO/TDO-product Kyn in the serum of patients receiving anti-PD-1 were recently reported as an adaptive resistance mechanism associated with worse overall survival^13,38^. Similarly, we here found that upregulation of Kyn-degrading enzymes correlates with good response to anti-PD-1 therapy (Fig. 1a). Therefore, identification of mechanisms that control the response to oncometabolites such as Kyn in the TME is likely to guide new therapeutic approaches. In this study, we provide evidence that the IDO/TDO-downstream AHR activation represents a common feature of cancers overexpressing IDO or TDO, which dictates a T cell suppressive TME through the establishment of a Treg-macrophage suppressive axis.

As reported previously, resistance to ICB therapy is commonly associated with high Treg/Teff ratio^39,40^ and strong immunosuppressive signatures conferred by macrophages, including angiogenesis and T cell suppressing soluble factors^41^. AHR is known to play an important role in modulation of immune responses and to be expressed by several cell types^42^. Our profiling of the TME indicates that Tregs and TAMs retain an immunosuppressive pro-tumoral function through the engagement of the AHR pathway in the presence of high IDO or TDO expression. A recent paper using a glioblastoma model showed an AHR-driven upregulation of the ectonucleotidase CD39 in TAMs which promoted T cell dysfunction^21^. In our study, changes detected in Tregs and TAMs were both quantitative and qualitative, as described in Figs. 2 and 3, and can account to the highly dysfunctional effector phenotype of CD8^+^ T cell in these IDO/TDO-expressing models. Although a causal role for low Trp levels in T cell inhibition cannot be fully discarded, the macrophage and Treg-dependent tumor progression detected in these models and its reversion upon AHR-blockade suggest that indirect TME-related mechanisms of T cell suppression drive IDO/TDO-mediated cancer progression through the AHR pathway.

In addition, our findings that an induction of AHR signaling in Tregs can modulate M2-like TAMs activity are interesting and reveal an undescribed role of AHR signaling in mediating immune resistance. Although the effects of AHR signaling can take place on multiple immune cells^43^, AHR activation in Tregs may be contributing to the establishment of a myeloid-enriched immunosuppressive TME. Other similar Treg-TAM interactions were shown in the context of cancer and other inflammatory diseases in recent publications, with Tregs promoting macrophage efferocytosis^30^ and accumulation of M2-like TAMs in the TME indirectly by limiting the secretion of the M1-inducer signal IFNγ in CD8^+^ T cells^29^. Although we identified AHR signaling as a key molecular mechanism controlling this Treg-macrophage interplay, further studies will be necessary to better elucidate this regulatory pathway.

In a previous report, activation of the AHR by tobacco smoke was able to induce PD-L1 expression and immune-escape in a lung cancer model^44^ and AHR activity predicted response to PD-L1 blockade. Here, we identified a common metabolic mechanism that participates in the control of Tregs, TAMs and T cell immunity in the context of IDO or TDO expression. Furthermore, we show that the efficient and selective targeting of the AHR pathway in tumors with an active Trp catabolic pathway overcomes immunosuppressive features in the TME and can sensitize otherwise immune resistant tumors to clinically approved PD-1 blockade. Our results provide a rationale for assessing active metabolic pathways in tumors, such as by monitoring the circulating levels of Kyn and Kyn/Trp ratio as recently reported^38^ in a precision-medicine type of approach, for the design of combination strategies using AHR inhibitors in future clinical trials.

## Methods

### Cell lines

The murine cancer cell lines for melanoma (B16F10, referred to as B16), and colon cancer (CT26) were obtained from ATCC. Cells were maintained in RPMI medium supplemented with 10% fetal calf serum (FCS) and penicillin with streptomycin (complete RPMI media). B16^IDO^ and B16^TDO^ cells were generated by transduction of B16F10 with GFP plus the IDO gene or TDO gene in the pCMV6-AC-GFP vector (Origene). B16F10 transduced with GFP alone were used as control cells (B16-WT). Clonal transformants were selected using 1 mg/ml G418 (Geneticin). Cell lines were routinely screened to avoid mycoplasma contamination and maintained in a humidified chamber with 5% CO_2_ at 37 °C for up to 1 week after thawing before injection in mice.

### Quantitative RT-PCR or mRNA expression profiling

Cells were lysed in TRIzol LS reagents (Life technologies) and frozen at −80 °C for future use. Process of total RNA extraction was performed according to the manufacturer’s instructions. Reverse transcription to cDNA was performed with 1 μg using a High Capacity cDNA Archive Kit (Life technologies). cDNA samples were pre-amplified using the PreAmp master mix (Fluidigm) and the mixtures of all the Taqman assays (lifetechnologies) to prepare preamplified cDNA by PCR (18 cycles), following the manufacturer’s instruction. Pre-amplified samples were loaded in a 48.48 dynamic array integrated fluidic circuit (IFC; Fluidigm) using an MX IFC controller (Fluidigm). mRNA expression data were processed by BioMark HD System and data analyzed using the real-time PCR analysis software (Fluidigm). mRNA levels were normalized to Gapdh and data were analyzed by applying the 2^(-dCt) calculation method. A list of primers can be found in Supplementary Table 1.

### Cytokine analysis

Tumors were excised, snap-frozen in dry ice, and pulverized using a tissue grinder. Tumor protein homogenates were prepared in PBS containing 2× Halt Protease and Phosphatase Inhibitor Cocktail (Fisher Scientific). Total protein concentration was normalized to 2 mg/ml. For cell supernatants, they were collected from cell culture, centrifuged at 4 C for 15 min at high speed to remove cells and frozen in −80 C freezer until the day of the experiment. Cytokines were quantified using the MILLIPLEX MAP Mouse Cytokine/Chemokine Magnetic Bead 32 Plex Panel according to the manufacturer’s instructions (Millipore, Billerica, Massachusetts).

### Mice

C57BL/6 and Balb/c mice (6–8-weeks old), Rag^−/−^ mice and CCR2^−/−^ on C57BL/6 background were purchased from Jackson Laboratory. Constitutive AHR knockout mice were obtained from Taconic. Pmel-1 TCR transgenic mice have been reported^45,46^ and were provided by N. Restifo (National Cancer Institute (NIH), Bethesda, MD). Foxp3^GFP^ knock-in mice were a gift from Dr. A. Rudensky (Memorial Sloan Kettering Cancer Center (MSKCC), New York, NY). Foxp3^DTR^ mice were generated in the laboratory of Dr. G. J. Hammerling (German Cancer Research Center (DKFZ), Heidelberg, Germany), and previously described^47^. All mice were maintained in microisolator cages and treated in accordance with the NIH and American Association of Laboratory Animal Care regulations. All mouse procedures and experiments for this study were approved by the MSKCC Institutional Animal Care and Use Committee.

### Tumor challenge and treatment experiments

On day 0 of the experiments, tumor cells were injected intradermally (i.d.) in the right flank. 2 × 10^5^ B16^WT^, B16^IDO^ or B16^TDO^ were injected into C57BL/6J mice. For studies in immune compromised mice, the study was done in C57Bl.6 *Rag1*^−*/*−^ mice. Mice were obtained from Jackson Labs and Charles River Labs. Treatments were given as single agents or in combinations with the following regimen for each drug. The AHR inhibitor drug CH-223191 was dissolved at 0.5% methyl cellulose (MC) and 0.5% Tween 80 in water and administered by oral gavage once a day at 50 mg/mg. Treatment was initiated on day 7 ending on day 21 post tumor implantation. Control groups received vehicle (0.5% MC, 0.5% Tween 80) without the active product. For Clofazimine, treatment was initiated on day 7 ending on day 21 post tumor implantation. Clofazimine was prepared in a solution of 5% DMSO in PBS and mice were treated with daily i.p. injections at 10 mg/kg as described^31^. Epacadostat was dissolved in a 0.5% MC vehicle solution and 680C91 in a 5% DMSO + 5% Solutol solution in water and administered daily by oral gavage at 300 mg/kg and 20 mg/kg, respectively. Anti-PD-1 antibody (250 μg per mouse, clone RPM1-14, Bio X cell) was injected intraperitoneally (i.p.) every 3 days, starting from day 3 after tumor implantation. Treatment with anti-CSF1R (BioXCell) at 300 μg/mouse and anti-CD8 (2.43) at 250 μg/mouse was started on day 3 post tumor challenge for every 3 days. Tumors were measured every second or third day with a calliper, and the tumor size was calculated based on an ellipsoid formula. Mice that had no visible and palpable tumors that could be measured on consecutive measurement days were considered complete regressions. Animals were euthanized for signs of distress or when the total tumor volume reached 2500 mm^3^. For the re-challenge study: mice with complete responses in the anti-PD-1 and CH-223191 combination group were re-challenged with 1 × 10^5^ B16-F10 tumor cells (on day 80 of the study since original tumor implant).

### AHR antagonism with KYN-101

A novel AHR antagonist, KYN-101 that is potent and selective inhibitor has been developed by Ikena Oncology, formerly known as Kyn Therapeutics. KYN-101 has an IC_50_ of 22 nM in the human HepG2 DRE-luciferase reporter assay and 23 nM in the murine Hepa1 Cyp-luc assay. For efficacy studies with B16-F10 melanoma model, C57Bl/6 female mice were inoculated intradermally in the hind flank with B16^IDO^ tumor cells at 2 × 10^5^ cells/mouse in a 50 μL injection volume. Seven days after cell inoculation, animals were randomized into 4 groups. Animals were dosed with vehicle, KYN-101, anti-PD1, or a combination of anti-PD1 and KYN-101. KYN-101 (10 mg/kg) and vehicle control (0.5% MC) were administered PO daily (QD) for a total of 12 doses. Anti-PD1 (250 μg/mouse) and Rat IgG control (in 1X PBS) were administered IP Q3D for a total of 4 doses. Tumor and body weight measurements were taken three times per week. Tumor volumes were calculated, percent inhibition of tumor growth and survival with KYN-101 as a single agent or in combination with anti-PD1 compared to vehicle control were determined. For the CT26 colorectal cancer model, Balb/C female mice were inoculated subcutaneously in the hind flank with CT26 tumor cells at 5 × 10^5^ cells/mouse in a 100 μL injection volume. Four days after cell inoculation, animals were randomized into 4 groups based on body weight. Animals were dosed with vehicle, KYN-101, anti-PD1, or a combination of anti-PD1 and KYN-101. KYN-101 (3 or 10 mg/kg) and vehicle control (0.5% MC) were administered PO daily (QD). Anti-PD1 (10 mg/kg) and Rat IgG control (in 1X PBS) were administered IP BIW for a total of 5 doses. Tumor and body weight measurements were taken three times per week. Tumor volumes were calculated and percent inhibition of tumor growth with KYN-101 as a single agent or in combination with anti-PD1 compared to vehicle control were determined.

### Kyn measurement

The biological activity of IDO and TDO was evaluated by measuring the level of L-kynurenine by ELISA according to the manufacturer’s instructions (LDN Labor Diagnostika Nord and Immundiagnostik). The conditioned medium related to the same number of cells was centrifuged at 12,000 × *g* for 5 min to remove debris. Samples were read with a SpectraMax 340PC (Molecular Devices) at 450 nm. Samples were read with a SpectraMax 340PC (Molecular Devices) at 450 nm.

### Purification of Tregs

Foxp3^+^ Tregs were purified from tumors of Foxp3GFP mice. Tumor single-cell suspensions were generated as described previously. CD4+ T cells were enriched by Ficoll gradient (Sigma-Aldrich), before sorting for GFP expression on a FACSAriaTM II Cell Sorter (BD Biosciences). Dead cells and doublets were excluded on the basis of forward and side scatter and Fixable Viability Dye eFluor 506. Purity of flow-sorted populations was above 90%.

### Cell depletions in mice

Foxp3^DTR^ mice were injected i.p. on days 7–8 and 14–15 after tumor implantation with DT (1 μg/mouse, Sigma-Aldrich). Clodronate liposomes were injected i.p. twice a week starting on day 3 after tumor implantation^48^.

### Isolation of tumor-infiltrating cells and lymphoid tissue cells

Tumor samples were minced with scissors before incubation with 1.67 U ml^−1^ Liberase (Roche) and 0.2 mg ml^−1^ DNase (Roche) in RPMI for 30 min at 37 °C. Samples were then processed by repeated pipetting and filtered through a 100-μm nylon filter (BD Biosciences) in RPMI to generate single-cell suspensions, which were subsequently washed with complete RPMI and purified on a Ficoll gradient to eliminate dead cells. Single-cell suspensions from spleens were obtained by grinding spleens through 40-μm filters. When required, samples were treated with red blood cell (RBC) lysis buffer (ACK Lysing Buffer, Lonza) and further washed and re-suspended in FACS buffer (PBS/0.5% albumin) before incubation with antibodies.

### Generation of bone-marrow derived myeloid cells and priming assay

Isolation and differentiation of bone marrow-derived antigen-presenting cells (BM-APCs) was performed as previously described^49^. In brief, bone marrow was obtained from mice femurs. After RBC lysis, the cells were plated in six-well plates with a density of 1 × 10^6^/ml in complete RPMI 1640 media supplemented with 25 ng/ml GM-CSF and treated with Kyn at 100 μM or DMSO. On days 3 and 6, nonadherent cells and 75% of culture media were exchanged for fresh media with or without Kyn treatment. A total of 80% of the cell population stained positive for CD11c and F4/80 by flow cytometry. For the T cell priming assay, on day 6 BM cells were cultured an additional day to maturity by exchanging 75% of the media, with the addition of 50 ng/ml LPS^28^. On day 7, antigen-presenting cells were pulsed with gp100 peptide and co-cultured for 48 hours with freshly isolated melanossomal antigen-specific CD8^+^ T cells (pmel) in a ratio of 10:1 (1 × 105/0.1 × 105) in a round-bottom 96-well plate (Corning). Pmel CD8^+^ T cells were isolated from the spleen of pmel mice by CD8 positive selection through MACS beads (Miltenyi Biotec), labeled with the proliferative tracker CFSE.

### Three-dimensional killing assay

The 3D collagen-fibrin gel-based killing assay has been previously described in depth^19^. For the isolation of intratumoral CD8^+^ T cells, B16^WT^ and B16^IDO^ tumors were excised on day 15 and dissected into single-cell suspensions, as described above. Tumor-isolated CD8^+^ T cells were enriched by Ficoll gradient (Sigma-Aldrich) before sorting as (CD45^+^TCRβ^+^CD8^+^) on a FACSAriaTM II Cell Sorter (BD Biosciences). In brief, 0.1 × 10^5^ viable B16-F10 target cells were co-embedded into collagen–fibrin gels with 1 × 10^5^ FACS-sorted effector CD8^+^ T cells in a 10:1 ratio (effector:target). CD8^+^ T cells isolated from the spleen of Pmel-1/gp100-specific TCR transgenic mice were used as a positive control. After 48 h of co-culture, collagen-fibrin gels were dissolved and target cells were diluted and plated into 6-well plates for a colony formation assay. After 7 days, cells were fixed using 3.7% formaldehyde and stained with 2% methylene blue before colony counting.

### T cell suppression assay

For the suppression assays with tumor-isolated Tregs, B16^WT^ and B16^IDO^ tumors were grown in Foxp3^GFP^ mice and Tregs (CD45^+^TCRβ^+^CD4^+^GFP^+^) sorted at day 15 after tumor implantation. Tregs were incubated with CellTrace Violet (CTV, Invitrogen)-labeled target CD8^+^ T cells immunomagnetically purified (CD8 Microbeads, Miltenyi Biotec) from spleens of CD45.1^+^ C57BL/6J congenic mice. Cultures were stimulated for 48–72 h with 0.5 μg/ml soluble αCD3 Ab and irradiated splenocytes before analyses of target CD8^+^ T-cell CTV dilution (proliferation) and CD44 upregulation (activation) by flow cytometry. For the suppression assays with TAMs, B16^WT^ and B16^IDO^ tumors were grown in C57BL/6J mice. At day 15 after tumor implantation, tumor-isolated macrophages (CD45^+^ CD11b^+^F4/80^hi^Ly6G^-^) were sorted at over 90% purity. CD8^+^ cells isolated from the spleen of naïve mice were purified using anti-CD8 (Ly-2) microbeads (Miltenyi Biotech) according to the manufacturer’s protocol. CD8^+^ T cells were then plated in complete RPMI media supplemented with 0.05 M β-mercaptoethanol onto round bottom 96-well plates (1 × 10^3^ cells per well) and were suboptimally stimulated with αCD3/αCD28 microbeads (Dynabeads Human T-Expander CD3/CD28, ThermoFisher). CD8^+^ T cells were plated with FACS-sorted TAMs at a ratio of 2:1 (E:T) for 48 h before analysis of PD-1 and CD44 upregulation (activation) by flow cytometry.

### Isolation of Pmel lymphocytes and adoptive transfer

Spleens and lymph nodes from pmel-1 TCR transgenic mice were isolated and grinded through 100 μM filters. After RBC lysis, CD8+ T cells were purified by positive selection using Miltenyi magnetic beads, The cells were labeled with CFSE and injected into recipient animals via tail vein as described in the figure scheme (Fig. 7d). The frequency and activation of pmel cells were measured 7 days after adoptive transfer of 1 × 10^6^ cells using Thy1.1 antibody.

### Co-culture Treg-macrophages

Tregs were isolated from spleens of Foxp3^GFP^ mice as described above. Cells were maintained with 2000 IU/mL of IL-2 (Peprotech) and stimulated with plate-bound αCD3 (1 μg/mL). Treatment was performed with Kyn (100 μM) and/or CH-223191 (10 μM) for 24 h prior to the start of co-culture with BMDMs, prepared as described above. Co-culture of pre-treated Tregs with BMDMs was performed for 24 h at a 1:1 ratio (1 × 10^5^ × 1 × 10^5^ cells). For the T cell priming assay, Tregs were isolated as CD4^+^CD25^+^ using MACS magnetic beads (Miltenyi) from the spleens and lymph nodes of female C57BL/6 mice or AHR^KO^ mice at 8-10 weeks of age. Frequency of FoxP3+ cells was over 85% post purification. BM-APCs on day 7 of GM-CSF maturation (as described above) were cultured in a 96-well round bottom plate coated with anti-CD3 (0.5 μg/mL) with CD8^+^ T cells on a 10:1 ratio (effector:target). WT or AHR^KO^ Tregs were plated on a 2:1 ratio with CD8^+^ T cells and treatments with Kyn (50 μM) and/or CH-223191 (5 μM) were added. Cells were analyzed 48 h later by flow cytometry.

### Flow cytometry and morphology analysis

Single cell suspensions obtained from mouse tumors and spleens were pre-incubated (15 min, 4 °C) with anti-CD16/32 monoclonal antibody (Fc block, clone 2.4G, BD Biosciences) to block nonspecific binding and then stained (30 min, 4 °C) with appropriate dilutions of various combinations of the following fluorochrome-conjugated antibodies (clone, dilution, supplier, catalog number): anti-CD206-PE (clone C068C2, 1:200, BioLegend, 141702), anti-MHC Class II (I-A/I-E)-eFluor 450 (clone M5/114.15.2, 1:200, eBioscience, 48-5321-82), anti-MHC Class II-APC-Cy7 (clone M5/114.15.2, 1:200, BioLegend, 107602), anti-CD44-Alexa 700 (clone IM7, 1:200, BioLegend, 103026), anti-PD-1-APC (clone RMP1-30, 1:200, eBioscience,17-9981-82), anti-CD11b-PE-TR (clone M1/70.15, 1:200, Invitrogen, 50-113-7581), anti-F4/80-BV650 (clone BM8, 1:200, BioLegend, 123149), anti-CD45-AF-700 (clone 30-F11, 1:200, eBioscience, 56-0451-82), anti-CD25-APC-Cy7(clone PC61, 1:200, BD Pharmingen, 557658), anti-CD11c-PE-Cy7 (HL3, 1:200, BD Pharmingen, 550283), anti-PD-L1-PE-Cy7 (clone 10F.9G2, 1:200, BioLegend, 124308), anti-CD86-APC (clone GL1, 1:200, BD Pharmingen, 558703), anti-MHC I (H-2Kd)-FITC (clone KH95, 1:200, BD Pharmingen, 553573), anti-TCRβ-APC (clone H57-597, 1:200, eBioscience, 17-5961-82), anti-CD4-BV650 (clone RM4-5, 1:200, BD Horizon, 563747), anti-CD8-PE-TR (clone 5H10, 1:200, Invitrogen, MCD0817), anti-Ly6C-PE-Cy7 (clone AL-21, 1:200, BD Pharmingen, 560593), anti-Thy1.1-PE (clone OX-7, 1:200, BioLegend, 202524), anti-Ly6G-PercP-Cy5.5 (clone 1A8, 1:200, BD Pharmingen, 560602). For intracellular stain, cells were further permeabilized using a FoxP3 Fixation and Permeabilization Kit (eBioscience) and stained with the antibodies: anti-AHR-PE (clone 4MEJJ, 1:100, eBioscience, 12-5925-82), anti-FoxP3-eFluor 450 (clone FJK-16s, 1:200, eBioscience, 48-5773-82), anti-Ki67-FITC (clone SolA15, 1:800, eBioscience, 11-5698-82), anti-T-bet-PE-Cy7 (clone eBio4B10 (4B10, 4-B10), 1:200, eBioscience, 25-5825-82), anti-Granzyme B-PECF594 (clone GB11, 1:50, BD Bioscience, 562462), anti-TGFβ-APC (clone 1D11, 1:200, R&D Systems, MAB1835-100), anti-Eomes-PE (clone Dan11mag, 1:200, eBioscience, 12-4875-82).Stained cells were acquired on a LSRII Flow Cytometer using BD FACSDiva software (BD Biosciences) and the data were processed using FlowJo software (Treestar). Dead cells and doublets were excluded on the basis of forward and side scatter and Fixable Viability Dye eFluor 506.

### Patient material

Patient samples were collected on a tissue-collection protocol approved by the institutional review board of the Memorial Sloan Kettering Cancer Center (IRB #06-107). Banked tissue samples were retrieved as part of the melanoma disease management biospecimen protocols (IRB# 00-144 and 19-101). We have obtained informed consent from all participants of this study for sample collection. Specimens from metastatic melanoma patients were processed within a period of 3 hours. Single cell suspensions from tumor samples were obtained by mincing the specimens into small pieces (1 mm^3^) and further dissociated using enzymatic digestion for 1 h at 37 °C, with constant shaking in a gentleMACS™ Octo Dissociator. Trypan Blue dye exclusion was used to determine cell viability.

TIL samples were washed and stained with appropriate dilutions of various combinations of antibodies and intracellular IDO staining was performed as described^2^. Melanoma cell suspensions were plated at 0.5e06 cells/mL in 96-well round bottom plates with treatment with KYN-101 at the concentrations indicated. Cells were acquired 24 h after for flow cytometry analysis and gene-expression analysis as described above.

### Bioinformatic analysis of clinical samples

To determine the expression of kynurenine catabolism enzymes (*KMO, KYNU* and *HAAO*), we downloaded the expression data from Riaz et al.^15^. We first calculated the log2 of each value, and then scaled the log2 expression values gene-wise to generate log2 and Z-scaled log2 values (“Log2” and “Z-Score” in figures, respectively). To determine the average expression of kynurenine degradation enzymes (KD Score), we calculated the mean Z-scaled log2 value of the three enzymes described above for each sample. Shown in the Fig. 1b are the “On Therapy” samples. For gene-expression analysis of AHR-related genes across immune subtypes in Fig. 1e, the normalized RNAseq expression matrices for the TCGA generated by the Pan Cancer Atlas initiative were downloaded [ref: https://gdc.cancer.gov/about-data/publications/pancanatlas] and the PanImmune Feature Matrix of Immune Characteristics generated were downloaded^16^ that identified and characterized six immune subtypes spanning the tumor types in the TCGA. The log-counts and metadata were subsequently processed in R and boxplots were made showing ranges of expression across the 6 immune subtypes for select genes using package ggplot. The Wilcoxon signed-rank test was subsequently used to assess any statistical significance in gene expression between immune subtypes. For the correlation analysis, mRNA expression levels were collected from cBioportal (http://www.cbioportal.org/) for skin cutaneous melanoma SKCM (TCGA, Provisional) (*n* = 472), lung squamous cell carcinoma LUSC (TCGA, Provisional) (*n* = 501), pancreas adenocarcinoma PADC (TCGA, Provisional) (*n* = 179) and DLBC (TCGA, *n* = 48) in April 2018. Spearman’s rank-order correlation were performed. For the IDO^high^ and IDO^low^ analysis, samples were divided into quartiles based on IDO1 expression and Spearman’s rank correlation analysis between *MRC1* and *FOXP3* was performed in the lowest and the highest *IDO1* quartile group in R with the *cor.test* function. For the survival analysis, patient survival data along with corresponding *CYP1B1* mRNA Expression Zscores were downloaded from cBioportal^50,51^ from the provisional Kidney Renal Clear Cell Carcinoma^52^ and Bladder Urothelial Carcinoma^53^ datasets. Patients from each dataset representing the low and high *CYP1B1* expression represent the bottom and top quartile, respectively, using the *quantile* function in R. Survival plots and log-rank statistical test were generated using *survfit* and *ggsurvplot* functions from the *survival* and *survminer* packages in R.

### Statistics and reproducibility

Where indicated, data were analyzed for statistical significance using Prism (GraphPad software, version 7.0) and reported as *P* values. Data were analyzed with a two-tailed Student’s t test when comparing means of two independent groups and one-way ANOVA when comparing more than two groups, as specified. *P* < 0.05 was considered statistically significant (*P < 0.05, **P < 0.01, ***P < 0.001, ****P < 0.0001). Evaluation of survival patterns in tumor-bearing mice was performed using the Kaplan–Meier method, and results were ranked according to the Mantel–Cox log-rank test. *P* < 0.05 was considered statistically significant. Survival was defined as mice with tumors <2500 cm^3^. Detailed information of the statistical test and number of replicates used in each experiment are appropriately reported in figure legend. All findings reported were reproducible and data shown are representative of at least two independent experiments, as specified, with comparable results in each experiment. The investigators were not blinded to allocation during experiments and outcome assessment.

### Reporting summary

Further information on research design is available in the Nature Research Reporting Summary linked to this article.

## Supplementary information

Supplementary Information

Reporting Summary

## Data Availability

The RNA-seq data used for correlation analysis and Kyn-degrading enzymes expression were publicly available [http://www.cbioportal.org/] and deposited in the National Center for Biotechnology Information (NCBI) databases under the following accession numbers: Gene Expression Omnibus (GEO): GSE91061; Sequence Read Archive (SRA): SRP094781; and BioProject: PRJNA356761. For gene-expression analysis of AHR-related genes across immune subtypes, the normalized RNAseq expression matrices for the TCGA generated by the Pan Cancer Atlas initiative were downloaded [https://gdc.cancer.gov/about-data/publications/pancanatlas]. All other relevant data are available in the article, supplementary information, or from the corresponding authors upon reasonable request.
