# Supplementary material for: Blockade of the AHR restricts a Treg-macrophage suppressive axis induced by L-Kynurenine
Source: Nat Commun. 2020 Aug 11;11:4011. doi: 10.1038/s41467-020-17750-z (PMC7419300; doi:10.1038/s41467-020-17750-z)
Supplement: Supplementary file 1 — Supplementary Information [file 41467_2020_17750_MOESM1_ESM.pdf]

Supplementary data

Blockade of the AHR restricts a Treg-Macrophage suppressive axis induced by L-Kynurenine

Campesato et al., 2020

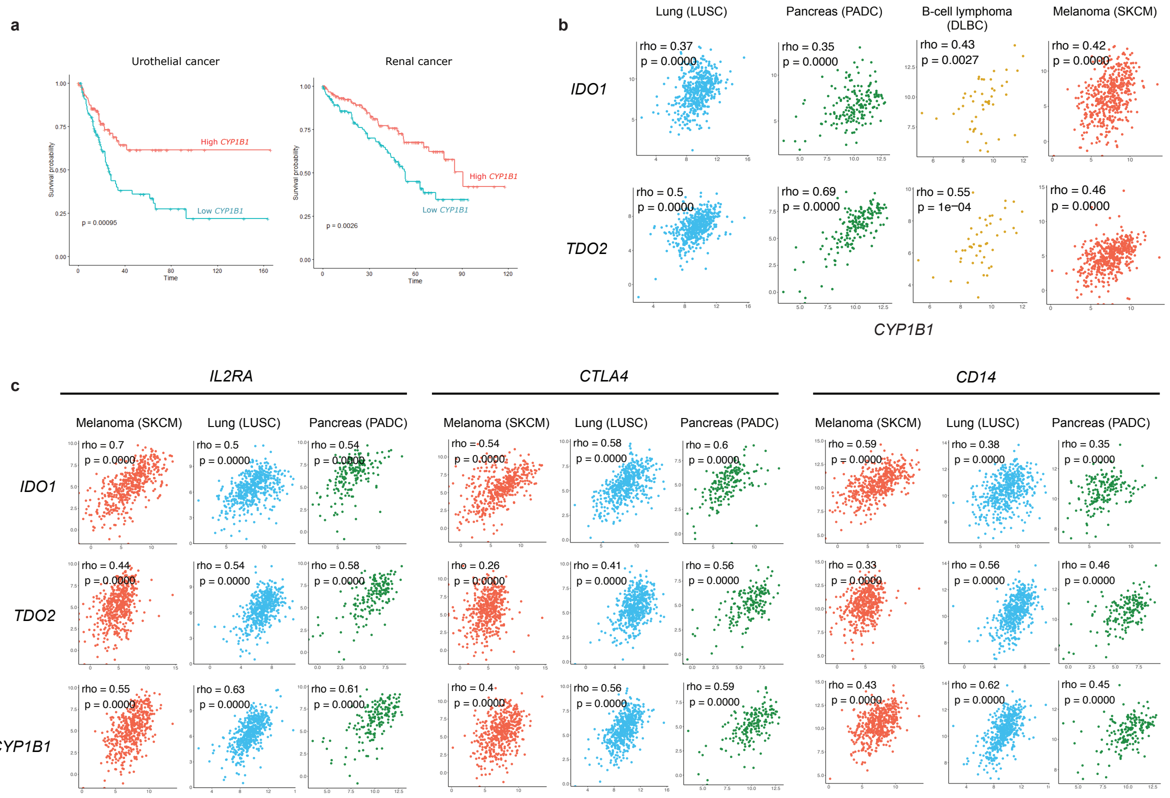

Supplementary Figure 1 | Expression of the AHR pathway in clinical samples associates with poor outcome. a, Survival probabilities of patients with urothelial cancer (n=406) or renal cell carcinoma (n= 510) with high expression (red) of *CYP1B1* (highest quartile) compared with those of patients with low (green) expression (lowest quartile, LogRank). Time in months. **b**, correlation between *IDO1*, *TDO2* and *CYP1B1* expression in TCGA RNAseq data of human melanoma (SKCM), human squamous lung cancer (LUSQ), human pancreatic adenocarcinoma (PADC) and diffuse large B-cell lymphoma (DLBC) analysed by Spearman rank correlation. **c**, correlation analysis between Treg marker (*IL2RA*), inhibitory checkpoint (*CTLA4*), myeloid-cell marker (*CD14*) and AHR-related genes *IDO1, TDO2* and *CYP1B1* in TCGA RNAseq data of skin melanoma (SKCM), squamous lung (LUSQ) and pancreatic adenocarcinoma (PDAC) analyzed by Spearman rank correlation.

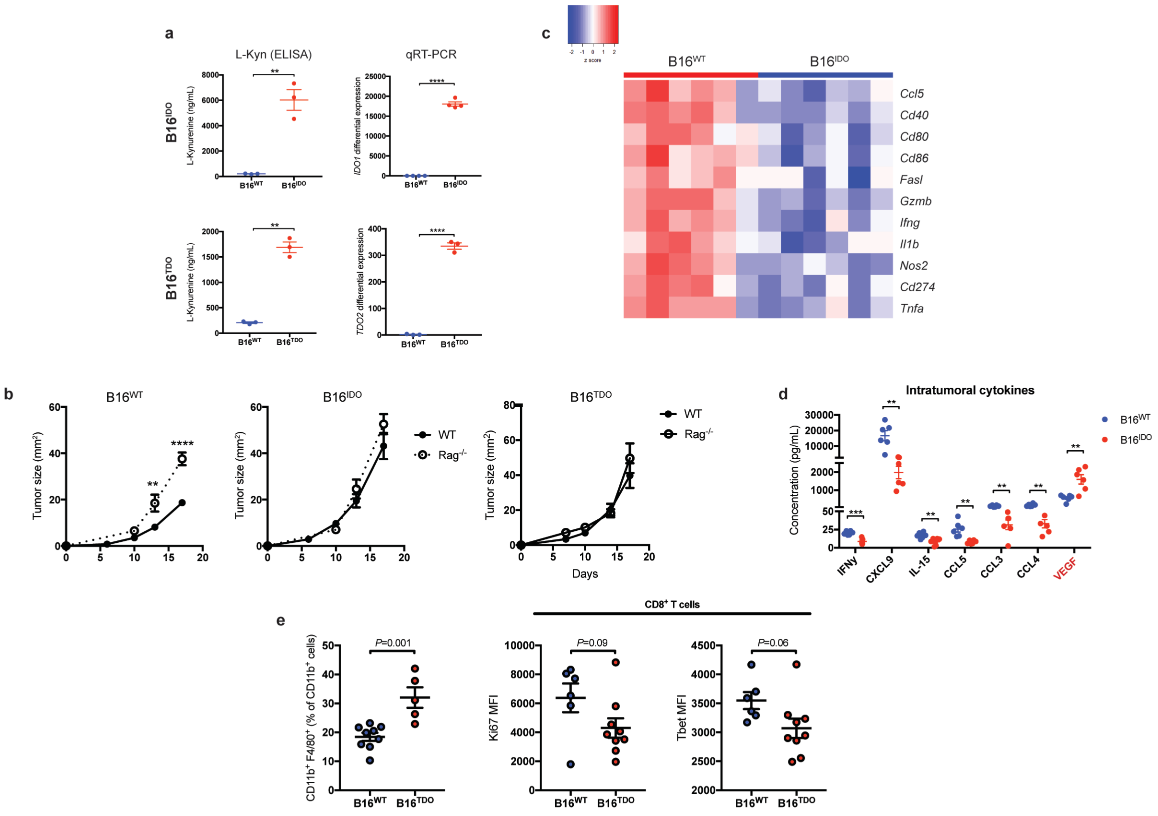

Supplementary Figure 2 | Overexpression of IDO/TDO in B16-F10 melanoma model leads to a distinct tumor immune activation status. a, ELISA of Kynurenine levels in cell culture supernatant (center) (n=6) and mRNA expression of *IDO1* and *TDO2* by qRT-PCR (right). (n=3) **b**, tumor progression of B16^{WT}, B16^{IDO} or B16^{TDO} implanted in immune-deficient (Rag$^{-/-}$). (n=10 WT and n=5 Rag$^{-/-}$). **c**, gene-expression analysis of B16^{WT} and B16^{IDO} whole-tumors by Fluidigm qRT-PCR analysis. **d**, Luminex analysis of cytokines in whole-tumor homogenates from B16^{WT} and B16^{IDO}. (n=6 per group). **e**, flow cytometry analysis of TAMs (CD11b^{+}F4/80^{hi}Ly6G^{-}) (left) and Ki67 and T-bet MFI in CD8^{+} T cells in B16^{WT} and B16^{TDO} tumors. Data represented as mean values ± SEM. Two-tailed unpaired Student's *t* test. P value: *P < 0.05, **P < 0.01, ***P < 0.001, ****P < 0.0001.

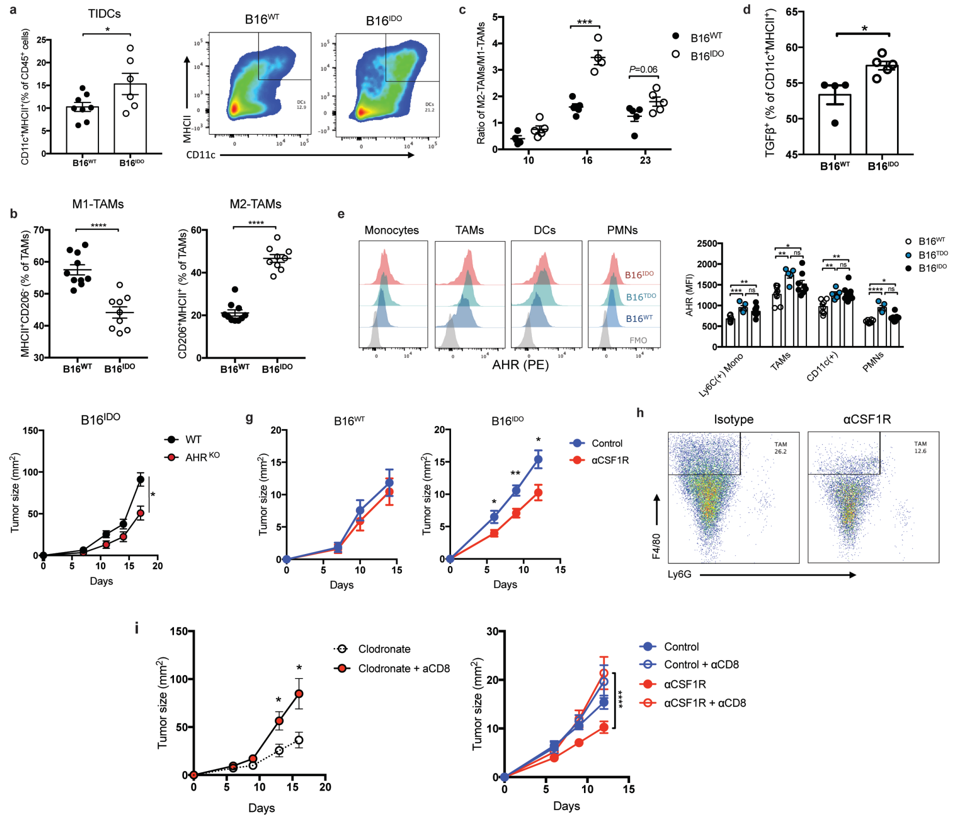

Supplementary Figure 3 | TAMs present enhanced AHR activity and tumor-promoting functions in IDO/TDO-expressing tumors. a, FACS analysis of dendritic cells (CD11c+MHCII+) in B16^{WT} and B16^{IDO} tumors. **b**, Ratio of M2-TAMs to M1-TAMs in in B16^{WT} and B16^{IDO} tumors over time. **c,** FACS analysis of TGFβ expression in tumor-infiltrating dendritic cells. **d**, FACS analysis of M1 (MHCII^{+}CD206^{-}) and M2 (CD206^{+}MHCII^{+}) TAM populations in B16^{WT} and B16^{IDO} tumor. **e**, flow cytometry histogram plots (left) and quantification (right) of AHR expression in tumor-infiltrating myeloid cell populations. **f**, tumor growth rate of B16^{IDO} in WT or AHR^{KO} hosts (n=10 per group). **g**, tumor growth rate (left) of B16^{WT} or B16^{IDO} treated with isotype or anti-CSF1R (300 μg/mouse) twice a week starting day 3 after tumor implantation (n=10 per group). **h**, Representative FACS plots (right) showing depletion of TAMs on day 7 after treatment. **i**, Tumor growth rate after treatment with clodronate liposomes (left) or anti-CSF1R (right) plus CD8 depleting antibodies (2.43) (left) (n=10 per group). Data represented as mean values ± SEM. Two-tailed unpaired Student's *t* test was used when only two groups were compared, and one-way ANOVA was applied with comparison of more than two groups. P value: *P < 0.05, **P < 0.01, ***P < 0.001, ****P < 0.0001.

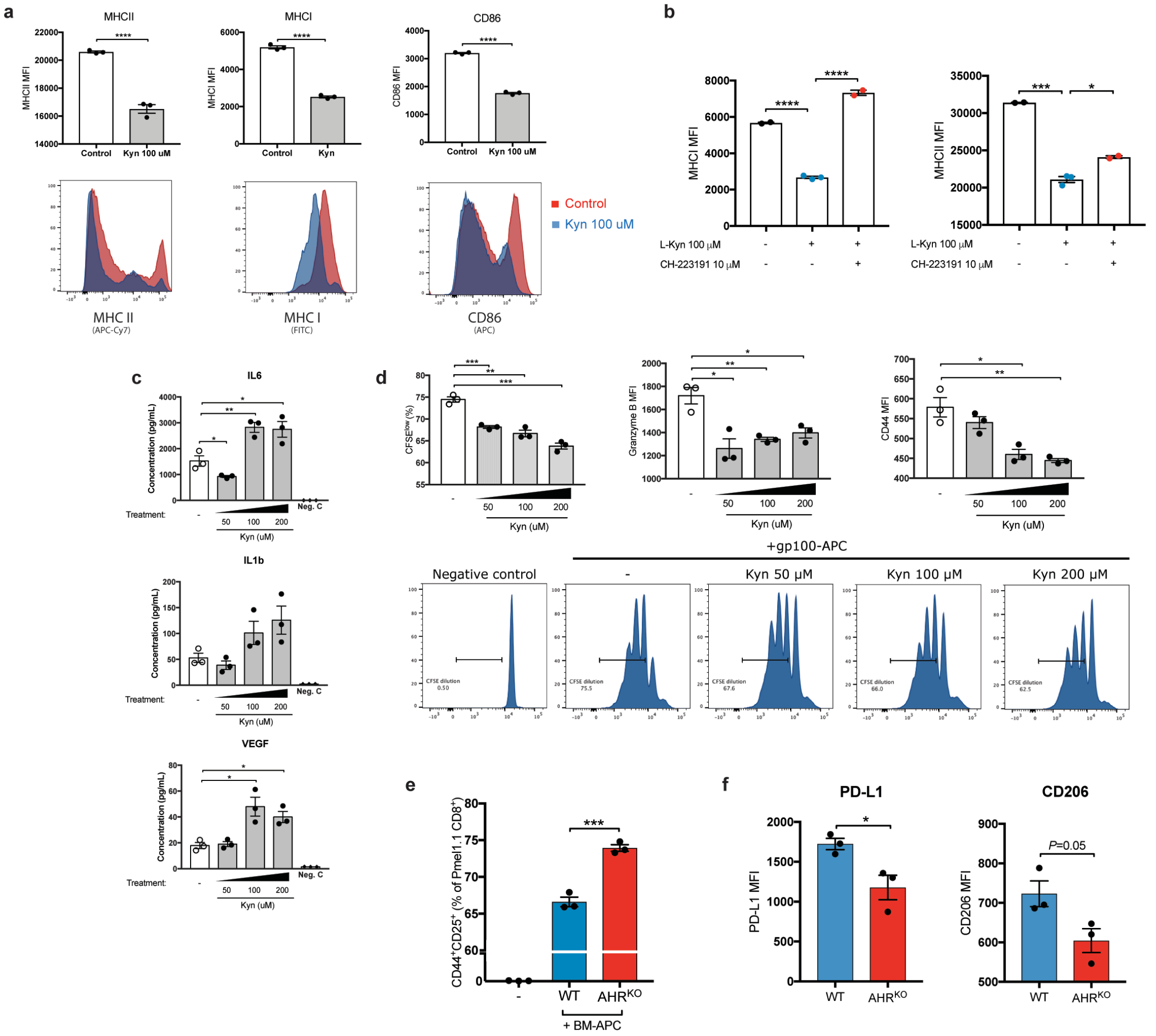

Supplementary Figure 4 | Modulation of the AHR pathway by IDO/TDO-derived L-Kyn impairs effector function of tumor-associated myeloid cells. **a**, FACS analysis and representative histograms of MHCII, MHCI and CD86 expression in bone-marrow derived macrophages treated with 100 μM of Kyn and **b**, with CH-22391. **c**, Luminex analysis of cytokines from bone-marrow derived macrophage cultures treated with increasing doses of Kyn. **d**, FACS analysis and quantification of proliferation markers (CFSE), Granzyme B and CD44 expression in $CD8^+$ T cells cultured in the presence of bone-marrow derived antigen-presenting cells (BM-APCs) treated with increasing doses of Kyn. **e**, FACS analysis of activation markers ($CD44^+CD25^+$) of CFSE-labeled Pmel1.1 $CD8^+$ T cells cultured in the presence of bone marrow derived APCs (BM-APCs) from wild-type (WT) or AHR^{KO} donors pulsed with gp100. aCD3/CD28 beads were used as positive control. **f**, FACS analysis of PD-L1 and CD206 MFI expression in BM-APCs. Data represented as mean values ± SEM. Two-tailed unpaired Student's *t* test. P value: *P < 0.05, **P < 0.01, ***P < 0.001, ****P < 0.0001.

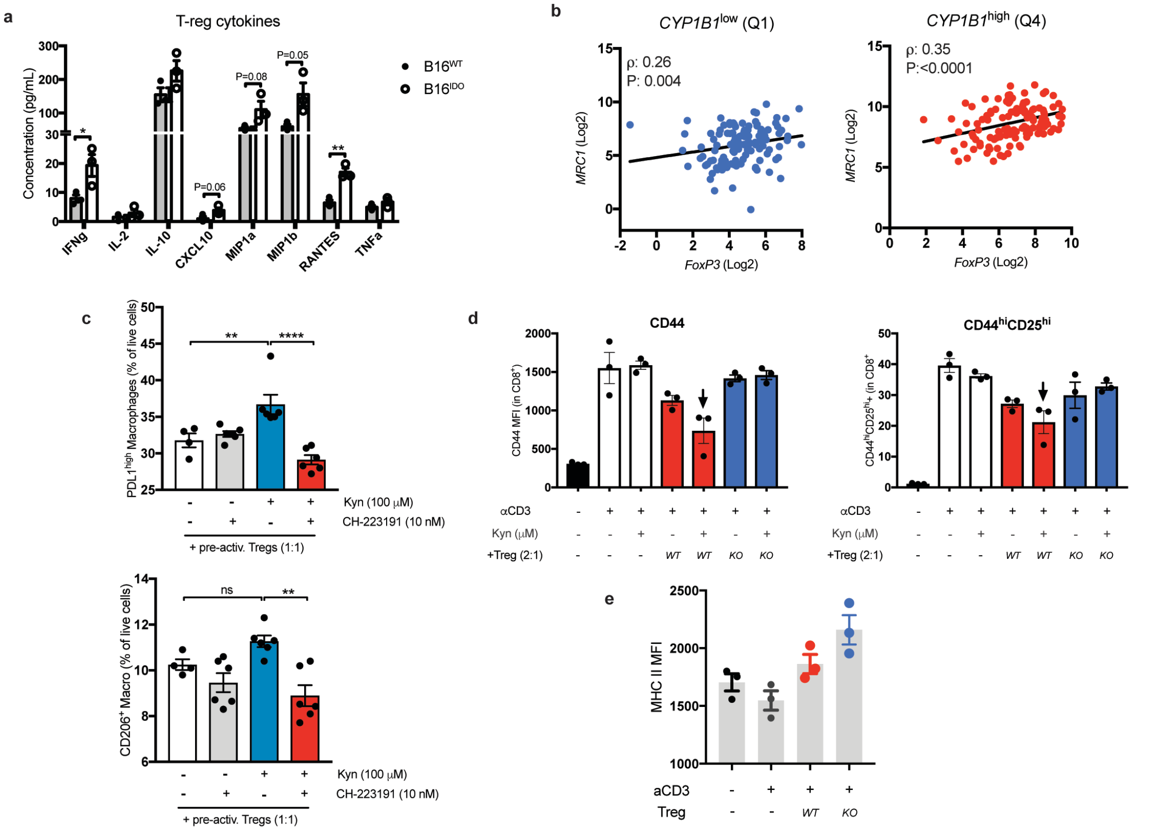

Supplementary Figure 5 | An active IDO-Kyn-AHR pathway mediates interactions between Tregs and macrophages. a, luminex analysis of cytokines from tumor-isolated Tregs ($FoxP3^{GFP+}$) after re-stimulation with aCD3/CD28 beads. **b**, correlation analysis between myeloid marker (MRC1/CD206) and Treg marker (FoxP3) in TCGA RNAseq data of skin melanoma (SKCM) $CYP1B1^{high}$ and $CYP1B1^{low}$ analyzed by Spearman rank correlation. **c**, FACS analysis of bone-marrow-derived macrophages co-cultured with splenic Tregs ($FoxP3^{GFP+}$) pre-treated with 100 µM Kyn (AHR agonist) and/or 10 µM Kyn CH-223191 (AHR antagonist) at a 1:1 ratio for 24h. **d**, FACS analysis of CD44 MFI in $CD8^{+}$ T cells (left) or frequency of $CD44^{high}CD25^{high}CD8^{+}$ (right) in a 48h co-culture with APCs and WT or KO-Tregs upon stimulus with Kyn (5:1:10/CD8:APC:Treg). Arrows indicate groups of Kyn-treated WT Tregs. **e**, FACS analysis of MFI of MHCII in APCs after co-culture with wild-type (WT) or AHR^{KO} (KO) Tregs. Data represented as mean values ± SEM. Two-tailed unpaired Student's *t* test was used when only two groups were compared, and one-way ANOVA was applied with comparison of more than two groups. P value: $^{*}P < 0.05$, $^{**}P < 0.01$, $^{***}P < 0.001$, $^{****}P < 0.0001$.

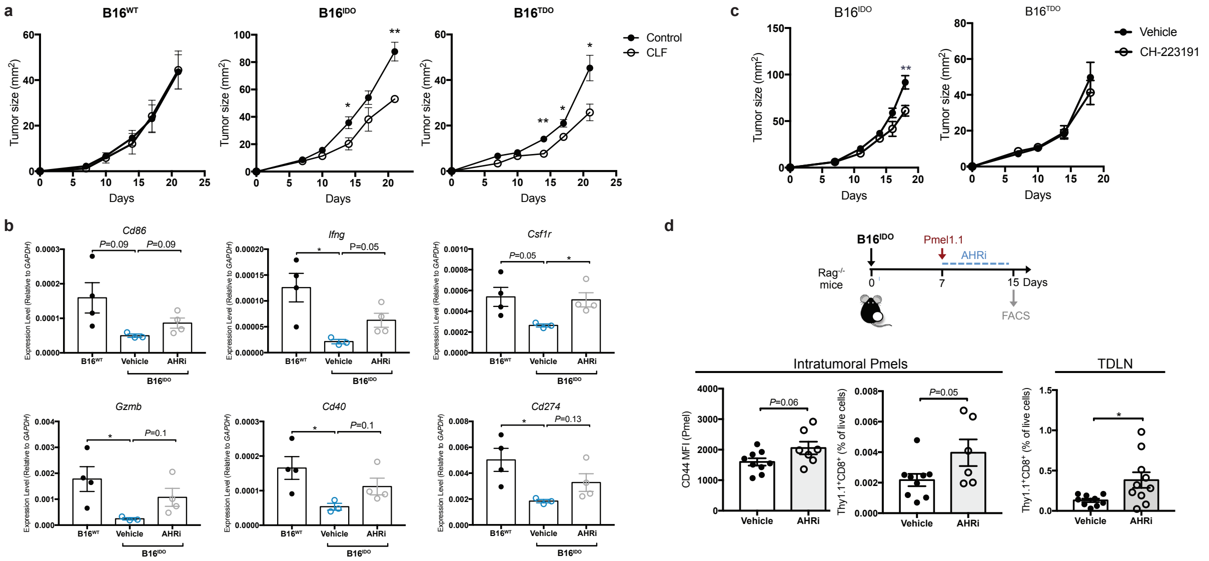

Supplementary Figure 6 | AHR inhibition promotes tumor control of IDO/TDO-expressing pre-clinical models. a, tumor size of orthotopically-injected B16^{WT}, B16^{IDO} and B16^{TDO} tumors in mice treated with daily i.p. injections of vehicle in PBS or CLF (10 mg/kg) (n=5 per group). **b**, mRNA analysis of whole-tumors by qRT-PCR after 7 days of treatment with vehicle or CH-223191 (50 mg/kg PO/QD) in B16^{WT}, B16^{IDO} and B16^{TDO} models. **c**, tumor size of B16^{IDO} and B16^{TDO}-bearing Rag$^{-/-}$ mice treated with vehicle or CH-223191 (50 mg/kg PO/QD) (n=5 per group). **d**, Representative graphs showing activation (CD44) and percentage Pmel1.1 of total CD8^{+} T cells in tumors and tumor-draining lymph node (TDLN) of control and AHRi-treated (CH-223191) B16^{IDO} tumor-bearing mice. Data represented as mean values ± SEM. Two-tailed unpaired Student's *t* test was used when only two groups were compared, and one-way ANOVA was applied with comparison of more than two groups. P value: $*P < 0.05$, $**P < 0.01$, $***P < 0.001$, $****P < 0.0001$.

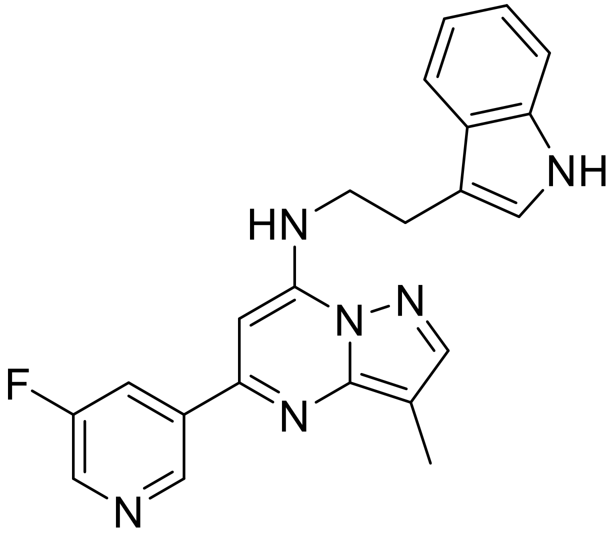

Supplementary Figure 7 | Chemical structure of KYN-101.

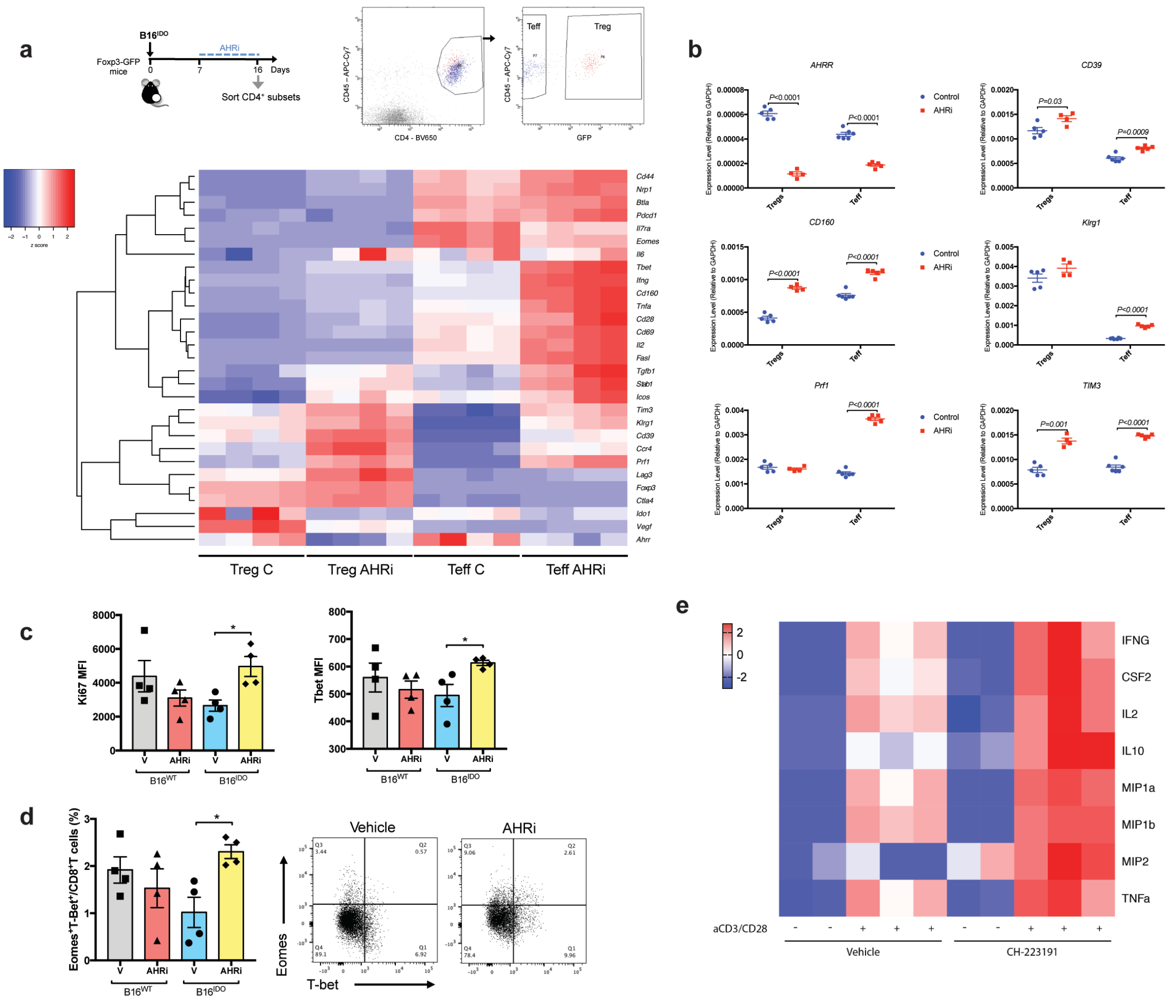

Supplementary Figure 8 | AHR inhibition promotes enhanced effector T cell function. a, Top, experimental scheme and FACS sorted CD4$^+$ T cell populations. Bottom, heat-map representing the gene-expression analysis of Treg and Teff-associated genes from CD4$^+$ populations isolated from B16^{IDO} tumors after AHRi treatment (CH-223191). **b**, mRNA of Treg and Teff-associated markers by qRT-PCR analysis in tumor-isolated Tregs (CD4$^+$GFP$^+$) or Teff (CD4$^+$GFP$^-$). **c**, flow cytometric analysis of Ki67 and T-Bet expression in CD8$^+$T cells and **d**, of Eomes$^+$T-Bet$^+$ CD8$^+$ T cells in B16^{WT}, B16^{IDO} models after treatment with vehicle or CH-223191 (50 mg/kg PO/QD). **e**, multiplex analysis of cytokines from ex-vivo stimulated (aCD3/CD28 beads) CD8$^+$ T cells from B16^{IDO} tumors treated with vehicle or CH-223191 (50 mg/kg PO/QD). Data represented as mean values ± SEM. Two-tailed unpaired Student's *t* test was used when only two groups were compared, and one-way ANOVA was applied with comparison of more than two groups. P value: *P < 0.05, **P < 0.01, ***P < 0.001, ****P < 0.0001.

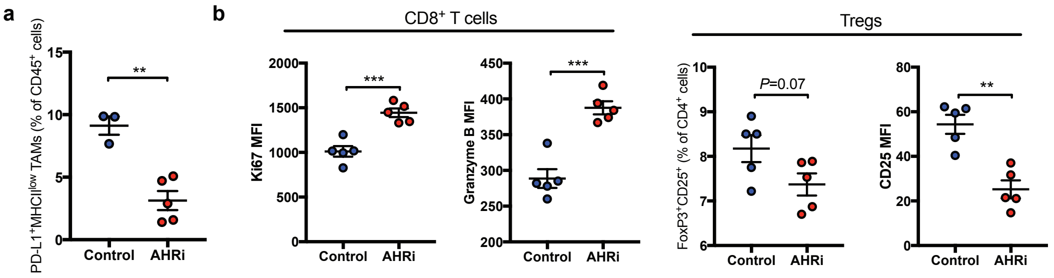

Supplementary Figure 9 | Immune modulation by AHR inhibition in IDO/TDO-expressing tumors. a, FACS analysis of M2-like TAMs (PD-L1$^+$MHCII^{low}) in B16^{TDO} tumors after treatment with AHRi (CH-223191) at 50 mg/kg/ PO/QD. **b**, FACS analysis of Ki67 and Granzyme B expression in CD8$^+$ T cell population (left); Treg frequency and CD25 expression (right) in tumor-draining lymph nodes of in B16^{TDO}-bearing mice after treatment with AHRi (CH-223191) at 50 mg/kg/ PO/QD. Data represented as mean values ± SEM. Two-tailed unpaired Student's *t* test. P value: *P < 0.05, **P < 0.01, ***P < 0.001, ****P < 0.0001.

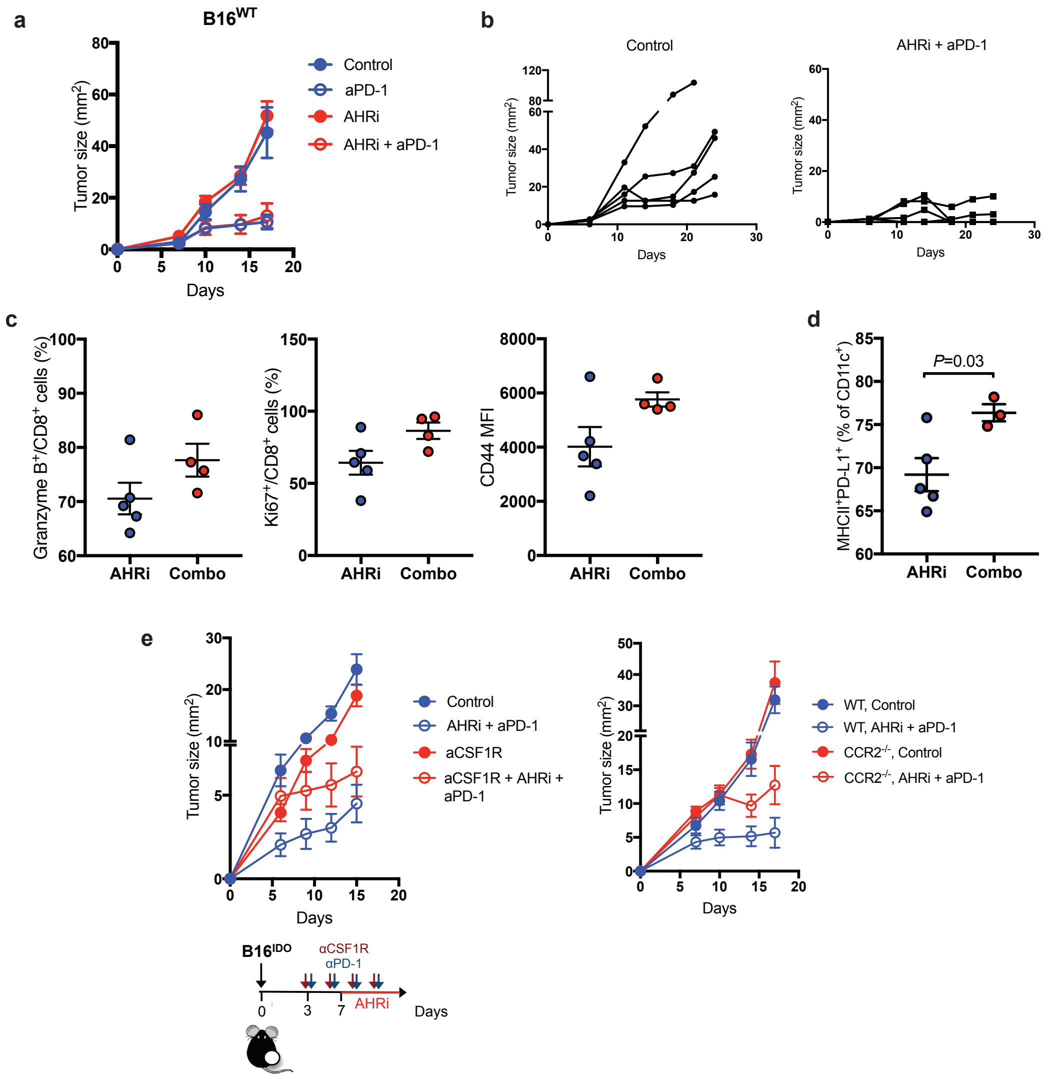

Supplementary Figure 10 | Combination of AHRi and anti-PD-1 promote enhanced antitumor immunity in a myeloid cell-dependent manner. a, mean tumor size of B16^{WT} tumor-bearing mice treated with AHR inhibitor (CH-223191), anti-PD-1 alone or in combination with AHR inhibitor (combo). (n=5 per group). **b**, tumor size of B16^{IDO} bearing mice previously treated with a combination of CH-223191 and aPD-1 (80 days after implantation) and re-challenged with 100.000 B16-F10 cells i.d. **c**, FACS analysis of activation markers (Granzyme B, Ki67 and CD44) in CD8^{+} T cells in B16^{IDO} tumors after treatment with AHRi (CH-223191) at 50 mg/kg/ PO/QD alone or in combination with anti-PD-1 (250 ug/mouse). **d,** FACS analysis of activated myeloid cells (MHCII^{+}PD-L1^{+}) in B16^{IDO} tumors after treatment with AHRi (CH-223191) at 50 mg/kg/ PO/QD alone or in combination with anti-PD-1 (250 ug/mouse). **e**, Tumor growth rate on B16^{IDO} tumors after treatment with AHRi (CH-223191) in combination with αPD-1 in myeloid cell-depleted mice (αCSF1R 300 ug/mouse or CCR2^{KO}) (n=10 per group). Data represented as mean values ± SEM. Two-tailed unpaired Student's *t* test was used when only two

groups were compared, and one-way ANOVA was applied with comparison of more than two groups. P value: *$P < 0.05$, **$P < 0.01$, ***$P < 0.001$, ****$P < 0.0001$.

Supplementary Table 1. List of primers for qRT-PCR

Gene name	Source	Identifier
Ahr	Thermo Fisher Scientific	Mm00478932_m1
Ahrr	Thermo Fisher Scientific	Mm00477443_m1
Btla	Thermo Fisher Scientific	Mm00616981_m1
Ccl5	Thermo Fisher Scientific	Mm01302428_m1
Ccr4	Thermo Fisher Scientific	Mm00438271_m1
Cd160	Thermo Fisher Scientific	Mm00444461_m1
CD274	Thermo Fisher Scientific	Mm00452054_m1<br>Hs00204257_m1
Cd28	Thermo Fisher Scientific	Mm01253994_m1
Cd39	Thermo Fisher Scientific	Mm00515447_m1
Cd40	Thermo Fisher Scientific	Mm00441895_m1
Cd44	Thermo Fisher Scientific	Mm01277163_m1
Cd69	Thermo Fisher Scientific	Mm01183378_m1
Cd80	Thermo Fisher Scientific	Mm00711660_m1
Cd86	Thermo Fisher Scientific	Mm00444543_m1
Ctla4	Thermo Fisher Scientific	Mm00486849_m1<br>Hs00175480_m1
Cyp1a1	Thermo Fisher Scientific	Mm00487218_m1
Cyp1b1	Thermo Fisher Scientific	Mm00487229_m1
Eomes	Thermo Fisher Scientific	Mm01351985_m1
Fasl	Thermo Fisher Scientific	Mm00438864_m1
Foxp3	Thermo Fisher Scientific	Mm00475156_m1
*Gzm*b	Thermo Fisher Scientific	Mm00442834_m1
Helios	Thermo Fisher Scientific	Mm00496108_m1
Icos	Thermo Fisher Scientific	Mm00497600_m1
Ido1	Thermo Fisher Scientific	Mm00492586_m1
Ifng	Thermo Fisher Scientific	Mm00801778_m1
Il10	Thermo Fisher Scientific	Mm00439616_m1<br>Hs00174086_m1
Il12	Thermo Fisher Scientific	Mm00434165_m1
Il1b	Thermo Fisher Scientific	Mm00434228_m1
Il2	Thermo Fisher Scientific	Mm00434256_m1
Il6	Thermo Fisher Scientific	Mm00446190_m1
Il7ra	Thermo Fisher Scientific	Mm00434295_m1
Klrg1	Thermo Fisher Scientific	Mm00516879_m1
Lag3	Thermo Fisher Scientific	Mm00493071_m1
Micb	Thermo Fisher Scientific	Hs00792952_m1
Nos2	Thermo Fisher Scientific	Mm00440485_m1
Nrp1	Thermo Fisher Scientific	Mm00435379_m1
Pdcd1	Thermo Fisher Scientific	Mm00435532_m1
Prf1	Thermo Fisher Scientific	Mm00812512_m1
Stab1	Thermo Fisher Scientific	Mm00460390_m1
Tbet	Thermo Fisher Scientific	Mm00450960_m1
Tgfb1	Thermo Fisher Scientific	Mm01178820_m1
Tim3	Thermo Fisher Scientific	Mm00454540_m1
Tnfa	Thermo Fisher Scientific	Mm00443258_m1
Vegf	Thermo Fisher Scientific	Mm00437304_m1
